# Gut microbiome dysbiosis and functional alterations in *Campylobacter*-associated gastroenteritis using metagenomic approaches

**DOI:** 10.1080/29933935.2026.2688065

**Published:** 2026-08-18

**Authors:** Bilal Djeghout, Alise J. Ponsero, Nuno Pedroso, George M. Savva, Ngozi Elumogo, Nicol Janecko

**Affiliations:** a Quadram Institute Bioscience, Norwich Research Park, Norwich, UK; b Centre for Microbial Interactions, Norwich Research Park, Norwich, UK; c Eastern Pathology Alliance, Norfolk and Norwich University Hospital, Norwich, UK

**Keywords:** Gastroenteritis, antimicrobial resistance, shotgun sequencing, microbiome, dysbiosis

## Abstract

*Campylobacter* species are a major cause of bacterial gastroenteritis worldwide. Using shotgun metagenomic sequencing of stool samples from PCR-confirmed *Campylobacter*-positive patients and symptomatic PCR-negative controls, we reveal dysbiosis marked by reduced species richness (median Shannon diversity was significantly lower in the *Campylobacter*-positive group [3.24] vs. *Campylobacter*-negative group [3.63], *P* = 0.038), taxonomic shifts toward inflammation-associated taxa (*Campylobacteriaceae*, *Enterobacteriaceae*, *Pasteurellaceae*), and depletion of key commensals involved in short-chain fatty acid (SCFA) production (*Ruminococcaceae*, *Bacteroidaceae*, *Eubacteriaceae*). These changes define a distinct microbial signature of infection, suggestive of a perturbed gut environment with reduced colonization resistance and impaired barrier function. Despite these taxonomic and ecological disruptions, resistome profiling showed no increase in the burden or diversity of antimicrobial resistance genes (ARGs), suggesting that the observed microbiome disruption may not lead to broader expansion of ARGs in the gut microbiome. Whole-genome sequencing of cultured *Campylobacter jejuni* and *C. coli* isolates revealed common ARGs, including *bla*_OXA-193_, *tet*(O), and *gyr*A_T86I, some of which overlapped with metagenomic findings. Moreover, metagenomics identified low-abundance *Campylobacter* species in PCR-negative controls, underscoring the need for greater taxonomic resolution. These results delineate a *Campylobacter*-associated microbial and functional footprint in the human gut, with implications for diagnostics and antimicrobial stewardship.

## Introduction

*Campylobacter* species are a leading global cause of bacterial gastroenteritis, resulting in substantial morbidity and economic burden.^1^ ^,^ ^2^ Infection, typically acquired through the consumption of contaminated food or water, manifests as an acute diarrheal illness and can, in some instances, lead to a systemic infection or cause chronic sequelae such as reactive arthritis and Guillain-Barré syndrome.^1^ ^,^ ^2^

The human gut microbiome, a complex ecosystem composed of trillions of microorganisms, plays a vital role in maintaining host health.^3-5^ The microbial community within each portion of the intestinal tract contributes to nutrient metabolism, immune system modulation, and colonization resistance against pathogens.^5^ A growing body of evidence highlights the role of the gut microbiome in shaping the human host response to *Campylobacter* infection and in influencing disease outcomes.^6-8^ Infections with *Campylobacter* can disrupt the gut equilibrium, inducing a state of dysbiosis characterized by altered microbial diversity, disturbed community structure, and impaired functional capacity.^9^ This disruption can compromise gut barrier integrity, promote mucosal inflammation, and increase susceptibility to secondary infections or post-infectious complications, including chronic sequelae.^10^ ^,^ ^11^

Characterizing the community composition and functional pathways affected by *Campylobacter* is critical for unraveling the mechanisms underlying *Campylobacter*-induced dysbiosis and for identifying potential targets for microbiome-based interventions.^3^ ^,^ ^9^ While previous studies have documented alterations in the gut microbiome associated with *Campylobacter* infection, a comprehensive understanding of the specific microbial shifts and functional changes is limited. In a longitudinal study of 24 poultry abattoir workers, fecal microbiota was profiled before, during, and after natural *Campylobacter* exposure using 16S rRNA sequencing, revealing persistent compositional shifts among individuals who became culture-positive, including increases in *Bacteroides* and *Escherichia* spp.^6^ Experimental animal models have also been used to characterize changes in gut microbiota during *Campylobacter* infection.^12^ In mice, *C. jejuni* colonization was accompanied by increased intestinal *E. coli* loads, while in chicken models, infection was associated with reductions in beneficial taxa such as *Lactobacillus* and *Corynebacterium* and expansions of *Streptococcus* and members of the *Ruminococcaceae* family. Although these systems enable controlled, mechanistic investigations, they may not fully recapitulate human gut microbiota composition or immune responses, and typically involve relatively small numbers of animals, limiting the extrapolation of results to natural human infection. Moreover, the effects of *Campylobacter* infection on antimicrobial resistance gene (ARG) profiles within the gut microbiome remain underexplored, despite growing concern about the dissemination of resistance in enteric pathogens.^13^ However, evidence from infections by other enteric pathogens indicates that such events can reshape the gut resistome. For instance, a metagenomic study of 60 patients with acute enteric infections (including *Campylobacter*, *Salmonella*, and *Shigella*) observed significantly higher diversity of ARGs in the acute phase compared to follow-up samples (*P* < 0.001); notably, *Escherichia* emerged as the principal carrier of ARGs, making up 20.9% of the total reads assigned to the genus *Proteobacteria*, with several extended-spectrum *β*-lactamases detected during infection that appeared diminished or transferred to different microbial hosts upon recovery.^13^ Incorporating comparative resistome profiling in *Campylobacter* infection could therefore reveal whether similar patterns exist, thereby filling a critical gap in our understanding of ARG dynamics in enteric infections. Notably, as the samples used in this study were obtained as anonymized residual diagnostic material, detailed clinical metadata, including antibiotic exposure history were not available; the resistome analysis presented here is therefore descriptive and associative rather than causal.

Shotgun metagenomic sequencing offers a powerful method to comprehensively characterize both taxonomic composition and functional potential of complex microbial communities, providing a higher resolution view of pathogen-associated microbiome alterations and resistome architecture.^14^ ^,^ ^15^ Applying this approach to *Campylobacter* gastroenteritis can identify specific microbial taxa and metabolic pathways perturbed during infection, elucidate mechanisms of microbial community disruption, and inform strategies to mitigate antimicrobial resistance and improve patient outcomes.^10^ ^,^ ^15^

The aim of this study is to characterize and compare the gut microbiome composition of stool samples with known *Campylobacter*-induced gastroenteritis and those with no known gastrointestinal infectious agents by shotgun metagenomic sequencing. We compare the microbial composition, diversity, and functional profiles of PCR-confirmed *Campylobacter* stool samples with those of PCR-negative stools selected to control for microbiome changes related to gastrointestinal symptoms rather than *Campylobacter* infection status alone. In addition to metagenomic profiling, we apply a culture-based approach to isolate *Campylobacter* strains from stool samples, enabling detailed genomic characterization of circulating isolates. Furthermore, we assess resistome profiles to evaluate ARG patterns associated with *Campylobacter* colonization. These analyzes aim to generate clinically relevant insights into *Campylobacter* infection-associated dysbiosis, antimicrobial resistance dynamics, and pathogen genomics, which are critical for advancing diagnostic and treatment approaches for *Campylobacter* gastroenteritis.

## Material and methods

### Stool sample collection

The study was approved by the University of East Anglia Research Ethics Committee (Reference: 2018/19-159). Residual diagnostic stool samples were accessed via the Norwich Research Park (NRP) Biorepository, an HTA-licensed tissue bank (HTA Licence No. 11208), under approval granted by the UK Health Research Authority (NRES Ref: 19/EE/0089; IRAS Project ID: 259062). All samples and associated metadata were fully anonymized prior to release to the research team. In accordance with the Human Tissue Act 2004 and UK biobanking governance frameworks, individual informed consent was not required for the use of anonymized residual diagnostic material supplied via the licensed biorepository. As part of biorepository governance procedures, checks against the NHS National Data Opt-Out database are performed to ensure that samples from individuals who have explicitly opted out of the use of their data or samples for research are not released.

Two separate sample collection periods were included: (i) July–September 2024 and (ii) May–July 2022 that were previously analyzed for a different set of metrics and published.^16^ From these collection periods, 60 stool samples comprising of: 30 *Campylobacter* PCR-positive and 30 PCR-negative were selected. PCR-negative samples were confirmed negative for the full enteric pathogen panel EntericBio Gastro Panel 2 (GP2, Serosetp, Ireland) that identifies *Salmonella*, *Shigella*, shiga toxin-producing *Escherichia coli*, *Campylobacter jejuni, C. coli, C. lari, Cryptosporidium parvum/hominis,* and *Giardia lamblia*. Both PCR-positive and PCR-negative samples were obtained from the biorepository of diagnostic samples submitted by patients presenting with gastrointestinal infection symptoms. Stool samples were submitted by general practitioners for routine diagnostic testing at the Eastern Pathology Alliance (EPA) microbiology diagnostic laboratory in Norwich, UK. Thus, the PCR-negative group comprises symptomatic individuals in whom these common enteric bacterial and parasitic pathogens were not detected, rather than healthy asymptomatic controls. No specific matching by age, sex, or other variables was conducted.

The sample inclusion criteria were: a sample volume of ≥5 mL, collection within 5 d of submission to the EPA laboratory, and stool consistency corresponding to a Bristol stool scale type of 5–7. Samples were selected consecutively from those available that met the inclusion criteria during the study period. The aliquots were transported under standard human tissue handling protocols to the Quadram Institute Bioscience (QIB) laboratory in Norwich, where they were processed for metagenomic DNA extraction and *Campylobacter* isolation. Basic anonymized patient metadata available for each sample included age group, sex, date of stool sample submission, and the clinical gastro panel 2 PCR results. Samples were obtained as anonymized residual diagnostic material from the biorepository; therefore, detailed clinical metadata, such as antibiotic exposure, symptom duration, comorbidities, and disease severity, were not within the scope of the study design.

### Campylobacter isolation from stool and whole genome sequencing

#### Microbiological culture

Each stool sample underwent *Campylobacter* isolation by direct culture using a modified ISO method (EN ISO 10272 - 2019) for detecting and enumerating *Campylobacter.* ^17^ In brief, a 10 μl aliquot of raw stool was directly plated onto a modified charcoal-cefoperazone deoxycholate agar (mCCDA) supplemented with cefoperazone and amphotericin-B (Oxoid, Hampshire, UK). All plates throughout the isolation protocol were incubated in a microaerophilic atmosphere using anaerobic jars with CampyGen 2.5 L sachet (Oxoid, Hampshire, United Kingdom) at 37 °C for 48 h. *C. jejuni* strain 81116^18^ was used as a positive control throughout the protocol. Once incubated, up to 20 suspected *Campylobacter* colonies per sample were subcultured onto a second mCCDA for purification, incubated, and further subcultured onto Columbia blood agar with 5% horse blood (Oxoid, Hampshire, UK). Typical *Campylobacter* colony morphology, microscopy, and oxidase testing (Thermo Fisher Scientific, Loughborough, UK) were used on presumptive *Campylobacter* isolates. A sample was classified as *Campylobacter* positive by culture if one or more presumptive isolates were confirmed as *Campylobacter* by sequenced taxonomic profiling. A total of 170 previously published^16^ *Campylobacter* genomes were included in this study.

#### Isolate DNA extraction and sequencing

Presumptive *Campylobacter* isolates underwent DNA extraction using Maxwell RSC Cultured Cells DNA Kits (Promega, Hampshire, UK) according to the manufacturer's instructions. DNA libraries were prepared using Illumina DNA Prep (Illumina Ltd., Cambridge, UK), as previously described^19^ and PE150 libraries were sequenced on Illumina's Nextseq500 instrument with a mid-output flowcell (NSQ® 500 Mid Output KT) (v2) (300 CYS) (Illumina, Cambridge, United Kingdom).

#### *Campylobacter* isolate genome analysis

Raw sequence reads of each sample were stored on an in-house instance of IRIDA.^20^ Illumina raw reads were trimmed using fastp (v0.19.5).^21^ Analysis was performed on the trimmed reads to predict bacterial genus and species and potential contaminations using Kraken2 (v2.1.1) with the prebuilt core_nt database (inclusive of GenBank, RefSeq, TPA, and PDB sequences; version 2024-09-04), downloaded on 5 February 2025.^22^ Kraken2 was selected for this purpose as its k-mer-based read classification approach provides rapid and sensitive species-level identification suitable for taxonomic verification and contamination detection in low-complexity isolate data. Paired-end reads were assembled using Spades (v3.12.0).^23^ The quality of the assemblies was assessed using QUAST (v5.0.2).^24^ Multi-locus sequence typing (MLST) (v 2.16.1)^25^ was conducted using PubMLST database (v2.16.1)^26^ for *C. jejuni* to assess sequence type (ST) on genome assemblies. AMR determinants and gene mutations were identified using AMRFinderPlus (v3.11.4).^27^ Phenotypic antimicrobial susceptibility testing was not performed in this study; antimicrobial resistance determinants were inferred from genomic data alone.

### Metagenomic DNA extraction and shot-gun sequencing

#### Stool sample preparation

To minimize the amount of human DNA accounting for sequencing capacity, a method of host DNA depletion was adapted for processing stool samples.^16^ ^,^ ^28^ In brief, 200 µl of buffer (5.5 M NaCl and 100 mM MgCl2 in nuclease-free water) was added to 200 µl of stool. This mixture was supplemented with 35 µl of 1% saponin (Tokyo Chemical Industry UK Ltd., Birkenhead, UK) and 10 µl of HL-SAN DNase (ArcticZymes, Tromsø, Norway). The samples were thoroughly mixed and incubated at 37 °C with shaking at 800 RPM for 20 minutes. Postincubation, the treated samples were washed with 300 µl of phosphate-buffered saline (PBS) (prepared using NaCl [58.44 g/mol], KCl [74.55 g/mol], Na2HPO4 [141.96 g/mol], and KH2PO4 [136.09 g/mol]). The samples were then centrifuged at 13,000 RPM for 5 min, and the supernatant was carefully removed to complete host DNA depletion.

#### Library preparation and sequencing of metagenomic DNA

Metagenomic DNA libraries were prepared using the Illumina DNA Prep (M) Tagmentation kit according to the manufacturer's protocol (Illumina Inc., San Diego, CA, USA). Paired-end indexed libraries were sequenced on the Illumina NovaSeq PE150 platform according to the manufacturer's guidelines (Novogene Cambridge, UK), with a target depth of at least 10 GB per sample. Quality control (QC) of the metagenome DNA libraries was performed prior to sequencing. This involved quantifying the DNA concentration using Qubit fluorometry and evaluating insert size and molar concentration with the Tapestation 4200 system using D5000 reagents and tape (Agilent, Santa Clara, USA).

### Metagenome data analysis

#### Sequences quality control

Initial quality control of the sequencing reads was performed using the QIB Clean-up pipeline v1.4 (https://github.com/telatin/cleanup). Potential human read contamination was further removed using Hostile (v1.1.0)^25^ against the human-t2t-hla reference database, and quality filtered using TrimGalore (v0.6.10).^29^ The complete pipeline used for the human decontamination and QC is available at: https://github.com/aponsero/QIB_WGScleanup. Sequencing depth varied significantly between sequencing runs, with samples from the run 2022 showing lower median read counts compared to those from 2024 (Wilcoxon test, *P* = 8.896e-05) (Figure S2A), The impact of the sequencing run on the microbiome richness and composition was systematically assessed in our alpha and beta-diversity analysis.

#### Community composition profiling

Taxonomic abundances within each metagenome were estimated using MetaPhlAn4 (v 4.0.1) against the Jun23_CHOCOPhlAnSGB_202307 database.^30^ MetaPhlAn4 was selected for community-level profiling of the stool metagenomes as its clade-specific marker gene approach normalizes for genome length and provides robust quantitative relative abundance estimates in complex microbial communities, outperforming k-mer-based classifiers for this task in independent benchmarking.^31^ The complete pipeline used for the taxonomic profiling is available at: https://github.com/aponsero/QIB_Metaphlan4.

#### Resistome analysis

Resistome analysis was conducted on the 60 metagenomic samples using ResFinder v2.4.0 with the GROOT algorithm^32^ to identify antimicrobial resistance (AMR) genes. Quality-filtered FASTQ reads were analyzed with a minimum identity threshold of 90% using Vsearch (v2.29)^33^ and rendered as a multiple sequence alignment file for indexing using GROOT. The generated index file was used to align all quality-controlled metagenomic reads, and a report was generated with a minimum gene cluster coverage of 90%. Gene read counts were normalized by gene length as reads per kilobase (RPK), and the resistome load was calculated by normalizing the RPK values by the total number of reads in each sample.

#### Statistical analysis

The community composition analysis was performed using the Microbiome R package (v1.24.0), and visualizations were generated using ggplot2 (v3.5). Ordination of the samples was performed using a PcoA on species level Bray–Curtis distances or Aitchison distances between samples, using the Microbiome R package.^31^ The CLR transformation in this package applies a pseudo count of min (relative abundance)/2 to exact zero relative abundance entries in the species abundance table before taking logs. PERMANOVA tests were implemented using the adonis2 function from the vegan package (v2.6.8) at the species level and included the sequencing depth and age of the participant as covariate when appropriate. Significant PERMANOVA results were systematically assessed using a beta-dispersion test.

The two study periods corresponded to two distinct sequencing runs. Because PCR-negative samples were sequenced in a single run and PCR-positive samples were distributed across both runs, the sequencing run was collinear with PCR status and could not be included as a covariate in between-group models. To assess whether the sequencing run introduced a technical batch effect, diversity and community composition were compared between the two sequencing runs within the PCR-positive group. Sequencing year was not significantly associated with any alpha-diversity metric (Figure S4) nor with species-level community composition in PCR-positive samples (PERMANOVA with 999 permutations, *P* > 0.05 for both the Bray‒Curtis and Aitchison metrics). These results indicate no evidence of a systematic batch effect, and no batch correction was applied.

#### Differential abundance analysis

Differential abundance testing was performed using MaAsLin2 (v1.16)^34^ to associate individual taxa with *Campylobacter* PCR status. MetaPhlAn4 relative abundances were used directly without renormalization after filtering of the unknown fraction. Linear models incorporated participant age (continuous), sequencing run (random effect), and *Campylobacter* PCR status as fixed effects. MaAsLin2 applied log transformation after adding pseudocounts (half the minimum abundance) to handle zero values. A minimum taxa abundance threshold of 0.1% and a minimum prevalence of 20% were applied. For antimicrobial resistance gene analysis, linear models were applied to count per million (CPM)-normalized log-transformed read counts using the same covariate structure.

## Results

### Cohort characteristics and microbiome analysis of *Campylobacter* PCR-positive and PCR-negative stool samples

#### Cohort composition and sequencing data quality

In this study, the cohort included 60 patients aged 9 months to 91 y (median: 59.5 y, IQR: 39.75–72 y), with 28 males and 32 females. The cohort was balanced between PCR-positive (*n* = 30) and PCR-negative (*n* = 30) groups for *Campylobacter* detection for comparative microbiome analyzes (Figure S1, Table S1). After QC and exclusion of human reads, the metagenomes had a median of 63 million read pairs, ranging from 0.98 million to 102 million read pairs (Figure S2). The proportion of reads annotated by MetaPhlAn4 at the Kingdom level was consistently high across all samples, with unclassified reads represented as empty segments. On average, 15.6% of the total reads were unclassified (Figure S2B). However, an outlier sample, m24EPA107N, a PCR-negative sample, showed a high proportion of unclassified reads (60%).

#### Bristol stool scores show no association with microbiome richness or composition

The Bristol stool scores, classifying samples to states of diarrhea composition were comparable between PCR-positive and PCR-negative groups (Chi-squared test, *P* = 0.96) (Figure S3A). The samples presented no significant association with microbiome species-level richness (Pearson correlation, coefficient = –0.1, *P* = 0.3) (Figure S3B) or microbiome species composition (PERMANOVA on species-level Bray‒Curtis distances, 999 permutations, *P* = 0.64) (Figure S3C). Owing to the consistent lack of association, Bristol stool scores were not included in the subsequent analyzes.

#### Age distribution and its impact on microbiome composition

The age of the cohort ranged from 9 months to 91 y, with similar age distributions between PCR groups (PCR-negative: median 66 y, IQR: 46.75–73.50; PCR-positive: median 55 y, IQR: 38.25–67.75; Wilcoxon test, *P* = 0.31) (Figure S3D). PCR-positive patients were predominantly aged 10–65 y, whereas the PCR-negative group included a higher proportion of older individuals. Notably, four PCR-negative samples were obtained from infants (<2 y) and young children (<8 y). Due to the absence of PCR-positive samples in these age ranges and the distinct gut microbiota composition in younger populations, the potential impact of excluding these samples was systematically assessed. Age was not significantly associated with species-level microbiome richness (linear regression, *P* = 0.21) (Figure S3E) but had a significant impact on microbiome species composition (PERMANOVA on species-level Bray‒Curtis distances, 999 permutations, *P* = 0.01, R² = 3.3%) (Figure S3F). These results remained consistent when excluding participants <10 y (richness: *P* = 0.36, composition: *P* = 0.042, R² = 2.9%). Age was systematically included as a covariate in subsequent models.

#### Sex distribution is imbalanced, but does not affect microbiome outcomes

Analysis of sex distribution revealed a significant imbalance between PCR groups, with more females in the PCR-negative group (19 females, 11 males) compared to the PCR-positive group (9 females, 21 males) (Chi-square, *P* = 0.01) (Figure S3G); however, the participant's sex was not significantly associated with microbiome richness (Wilcoxon rank-sum, *P* = 0.17) (Figure S3H) nor did sex significantly affect microbiome species composition (PERMANOVA on species-level Bray‒Curtis distances, 999 permutations, *P* = 0.174) (Figure S3I); therefore, it was not included in the following analyzes.

#### Culture recovers *C. jejuni* and *C. coli* from most PCR-positive samples, revealing diverse sequence types and antimicrobial resistance genes

Out of 30 PCR-positive stool samples tested for *Campylobacter*, 20 samples yielded isolates by culture (Table S2). Samples collected in 2022 and 2024 showed broadly similar species distributions and AMR profiles (Table S2). A total of 243 *Campylobacter* isolates were recovered from the 20 culture-positive samples, and the number of isolates per sample ranged from 3 to 20 (Table S2). Among these, 222 isolates (91.4%) were identified as *C. jejuni* and 21 isolates (8.6%) as *C. coli*. *C. jejuni* isolates were categorized into 12 distinct sequence types (ST) (Table S2). *C. coli* isolates from two different samples were identified as ST-1055 and ST-825. Two samples had more than one *C. jejuni* ST present (Table S2). At least one AMR determinant gene was detected in the majority of *C. jejuni* isolates, with *bla*_OXA-193_, *tet*(O), and *gyr*A_T86I being the most common AMR determinants. In *C. coli* isolates, *tet*(O) was identified in isolates from ST-1055. Several *C. jejuni* isolates also carried additional resistance determinants, including *50S_L22_A103V*, *bla*_OXA-447_, *gyr*A_P104S, and *aad*E (Table S2).

#### Assessment of the microbial community composition across *Campylobacter* PCR-positive and PCR-negative stool samples

##### Reduced species richness observed in *Campylobacter* PCR-positive samples.

**Figure 1.**
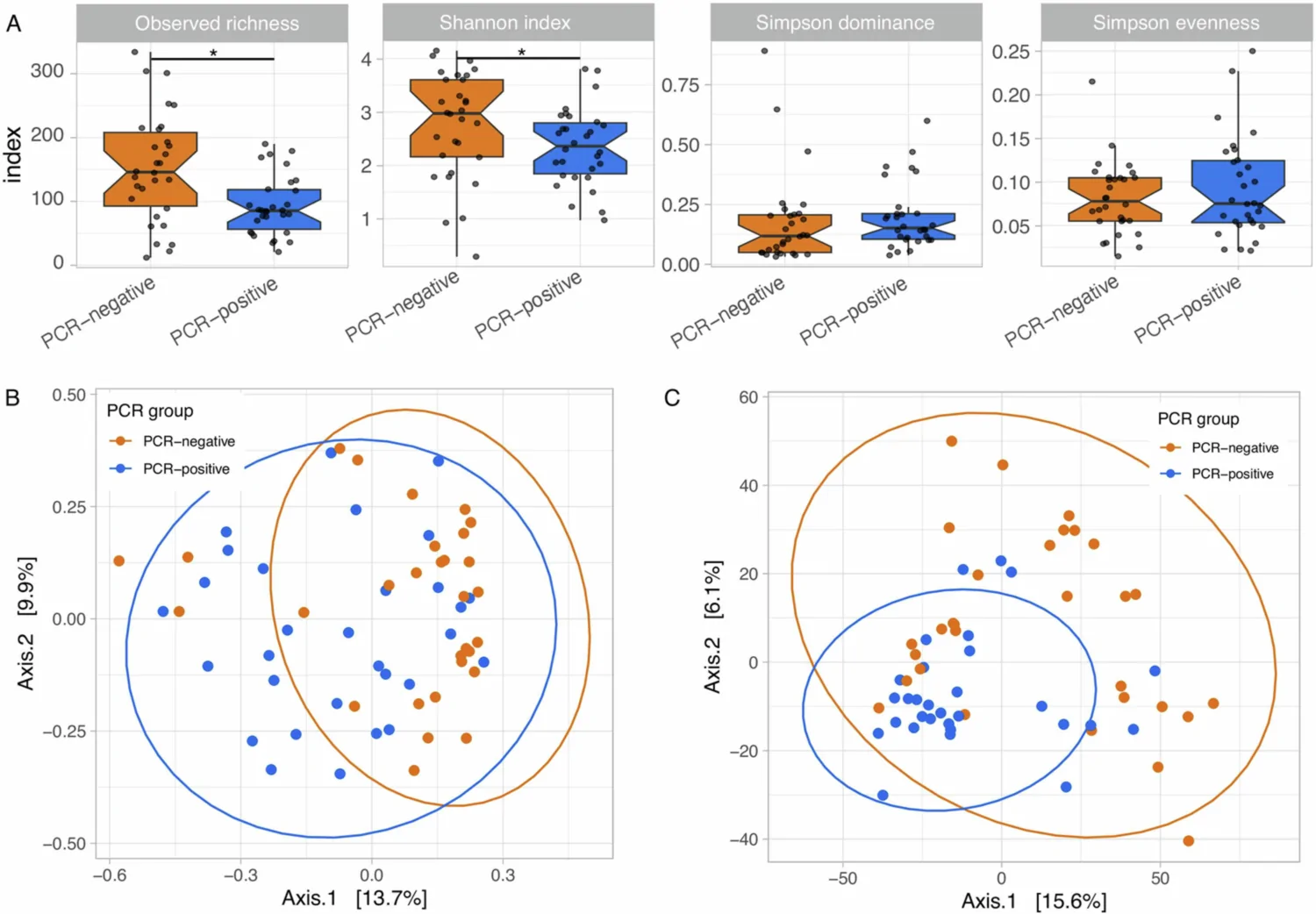
Difference in diversity and composition between PCR-positive and PCR-negative stool samples. (A) Diversity indices (species richness, Shannon index, Simpson dominance, and Simpson evenness) in PCR-positive and PCR-negative groups. Notches represent 95% confidence intervals. Notches represent 95% confidence intervals of the median. All panels: group comparisons were performed using linear models adjusting for age and sequencing depth. Significance codes: *** *P* < 0.001, ** *P* < 0.01, * *P* < 0.05. (B–C) Principal coordinate analysis (PCoA) of species-level beta diversity based on B) Bray–Curtis dissimilarity or C) Aitchison distances. For the second distance, species-level counts were center log-ratio (CLR) transformed after the addition of a pseudo-count. Samples are coloured by PCR group, with ellipses representing 95% confidence intervals.

At the species level, microbiome richness was significantly lower in the PCR-positive group (median: 85.5, IQR: 56.5–118.5) than in the PCR-negative group (median: 146, IQR: 92.75–208), after adjusting for age and sequencing depth (linear model, *P* = 0.014). Shannon diversity index was also significantly lower in PCR-positive samples (median: 2.36, IQR: 1.84–2.79) compared to PCR-negative samples (median: 2.97, IQR: 2.16–3.6) (*P* = 0.035), whereas no significant differences were observed for Simpson dominance (PCR-positive median: 0.152, PCR-negative median: 0.118; *P* = 0.32) or Simpson evenness (PCR-positive median: 0.075, PCR-negative median: 0.078; *P* = 0.28) (Figure 1A). Results remained consistent when excluding participants <10 y (richness: *P* = 0.004, Shannon: *P* = 0.021, dominance: *P* = 0.33, evenness: *P* = 0.50).

##### Significant shifts in overall community composition associated with *Campylobacter* infection.

Potential effects of PCR groups on global microbial community composition at the species level were assessed using Bray–Curtis and Aitchison dissimilarity metrics, adjusting for age and sequencing depth. The PCR groups showed significant differences in community composition for both the Bray–Curtis metric (PERMANOVA, 999 permutations, *P* = 0.016, R² = 3.1%) (Figure 1B) and the Aitchison metric (PERMANOVA, 999 permutations, *P* = 0.004, R² = 3.5%) (Figure 1C). Beta-dispersion analysis indicated no significant differences between groups for the Bray–Curtis distance (ANOVA, *P* = 0.2), whereas significant differences were observed for the Aitchison distance (ANOVA, *P* = 0.0005). Results remained consistent when excluding participants <10 y (Bray–Curtis: *P* = 0.012, R² = 3.2%; Aitchison: *P* = 0.003, R² = 3.8%).

##### Family-level microbial profiles differentiate PCR-positive and PCR-negative groups.

**Figure 2.**
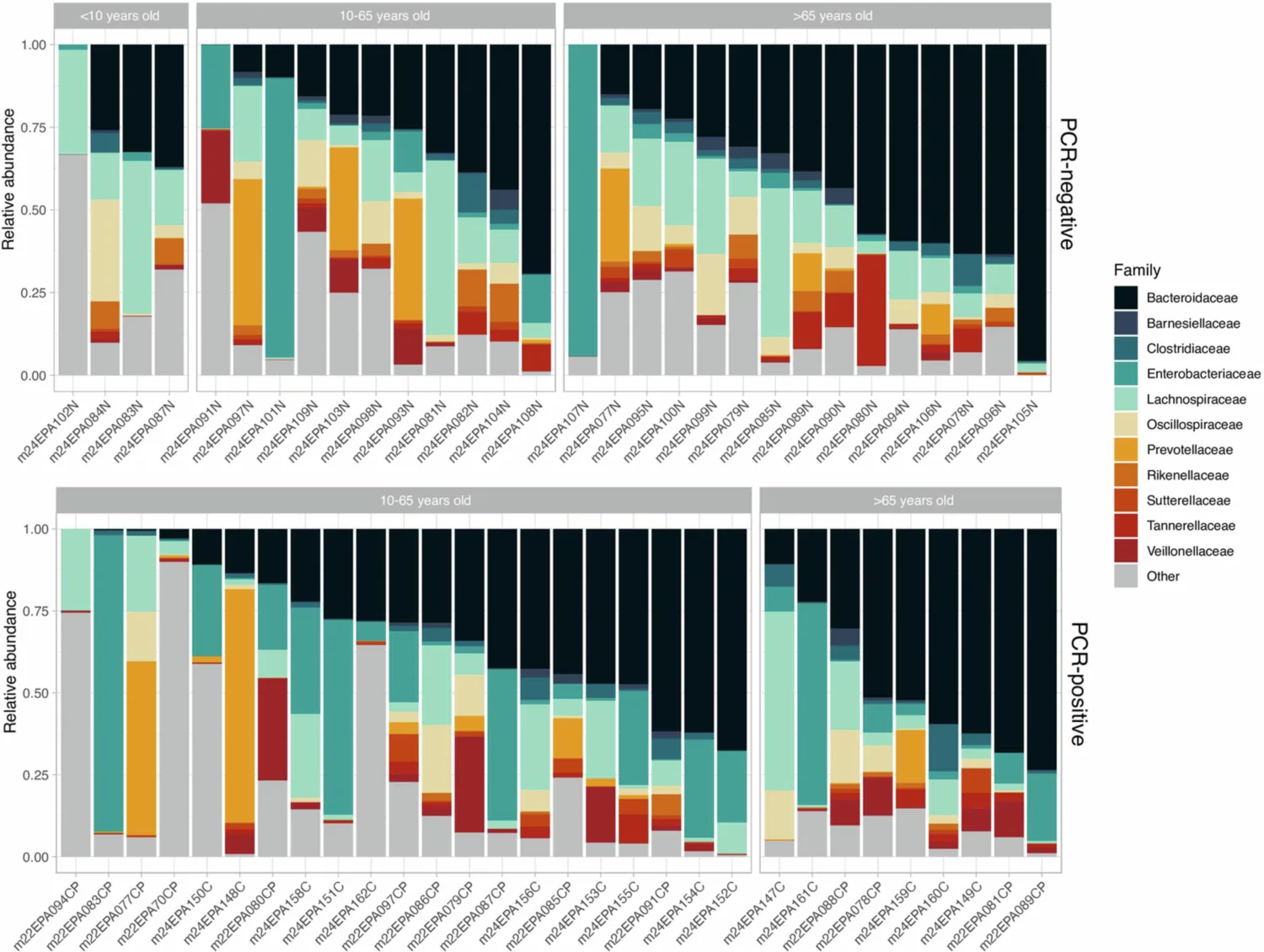
Family-level stool microbiome composition in PCR-positive and PCR-negative patient groups. Family-level microbiome composition of stool samples stratified by age category and PCR status. Relative abundances were aggregated at the family level; families with a prevalence below 20% and relative abundance below 1% were grouped as “Other.”

We next investigated the overall microbiome composition across the cohort, which showed characteristics of the human gut microbiome during gastrointestinal illness (Figure 2). At the family level, *Bacteroidaceae* dominated the communities with a median relative abundance of 27.3% (IQR: 14.1%–43.6%). *Lachnospiraceae*, represented the second most abundant group at 7.3% (IQR: 2.6%–17.5%), while *Enterobacteriaceae*, often associated with inflammatory conditions, showed a median abundance of 1.8% (IQR: 0.5%–13.1%) with notably high variability across samples. Other families included *Oscillospiraceae* (median: 1.8%, IQR: 0.1%–5.6%), *Clostridiaceae* (median: 1.2%, IQR: 0.2%–2.8%), *Tannerellaceae* (median: 0.9%, IQR: 0.004%–2.1%) and *Sutterellaceae* (median: 0.3%, IQR: 0.0%–1.1%). The high variability observed across most taxa (large IQRs relative to medians) reflects the heterogeneous nature of gut microbiomes during enteric illness.

At the genus level, the *Bacteroidota* phylum was represented primarily by *Phocaeicola* (median: 11.9%, IQR: 5.2%–19.3%) and *Bacteroides* (median: 11.2%, IQR: 5.6%–28.3%), both of which are important gut commensals. *Escherichia* showed considerable abundance variation (median: 1.6%, IQR: 0.2%–11.0%), likely reflecting its facultative pathogen status during gut inflammation. SCFA-producing genera were also detected, including *Mediterraneibacter* (median: 1.4%, IQR: 0.4%–3.5%), *Clostridium* (median: 0.7%, IQR: 0.06%–1.5%), and *Faecalibacterium* (median: 0.4%, IQR: 0.0%–2.2%), though with generally lower abundances and high inter-individual variation typical of dysbiotic states.

##### Increased abundance of *Campylobacteriaceae*, *Pasteurellaceae*, and *Enterobacteriaceae* in PCR-positive samples.

**Figure 3.**
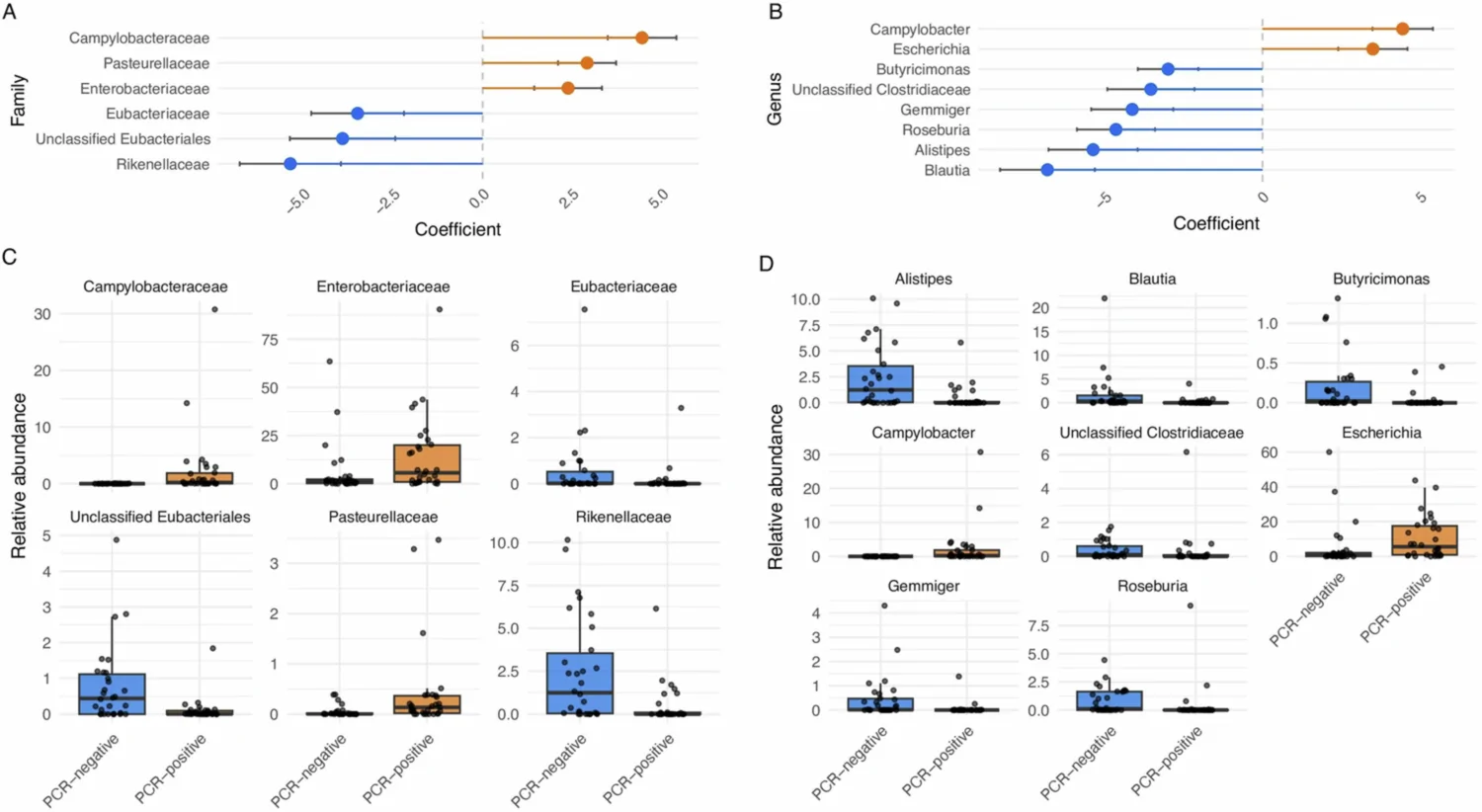
Differentially abundant taxa in PCR-positive and negative samples. (A) Differential abundance of microbial families between PCR-positive and PCR-negative groups. The lollipop plot shows families with significant differences in abundance between PCR groups, as determined by MaAsLin2 analysis adjusting for age and sequencing run (minimum abundance threshold: 0.1%, minimum prevalence: 20%, q-value < 0.1). Points represent regression coefficients (log-fold changes) for each taxon, with error bars indicating standard error. Positive coefficients indicate higher abundance in the PCR-positive group, whereas negative coefficients indicate higher abundance in the PCR-negative group. (B) Distribution of relative abundances for significantly different families. Box plots show the median (center line), interquartile range (box), and 1.5 times the interquartile range (whiskers), with individual sample points overlaid. Samples are grouped by PCR status, with the same color scheme as panel A. (C) Differential abundance of microbial genera between PCR-positive and PCR-negative groups, analyzed using the same MaAsLin2 parameters as panel A. Points represent regression coefficients (log-fold changes) for each genus, with error bars indicating standard error. (D) Distribution of relative abundances for significantly different genera. Box plots show the median (center line), interquartile range (box), and 1.5 times the interquartile range (whiskers), with individual sample points overlaid.

Differential abundance testing, adjusting for age and sequencing run, revealed significantly increased relative abundance of several families in the PCR-positive group: *Campylobacteraceae* (PCR-positive median: 0.206%, IQR: 0.023–1.872; PCR-negative median: 0.000%, IQR: 0.000–0.001; q = 0.0009), *Pasteurellaceae* (PCR-positive: 0.135%, IQR: 0.019–0.365; PCR-negative: 0.003%, IQR: 0.000–0.019; q = 0.048), and *Enterobacteriaceae* (PCR-positive: 5.609%, IQR: 0.905–19.987; PCR-negative: 1.301%, IQR: 0.179–2.279; q = 0.085). Conversely, *Rikenellaceae* (PCR-positive: 0.000%, IQR: 0.000–0.099; PCR-negative: 1.244%, IQR: 0.039–3.554; q = 0.008) and *Eubacteriaceae* (PCR-positive: 0.000%, IQR: 0.000–0.007; PCR-negative: 0.013%, IQR: 0.000–0.521; q = 0.048) were significantly depleted (Figure 3).

At the genus level, *Campylobacter* (q = 0.001) and *Escherichia* (PCR-positive: 5.609%, IQR: 0.905–17.543; PCR-negative: 0.633%, IQR: 0.047–2.081; q = 0.033) showed significantly higher relative abundances in the PCR-positive group. Conversely, multiple genera were significantly depleted, including *Blautia* (PCR-positive: 0.000%, IQR: 0.000–0.014; PCR-negative: 0.323%, IQR: 0.008–1.532; q = 0.001), *Alistipes* (PCR-positive: 0.000%, IQR: 0.000–0.099; PCR-negative: 1.244%, IQR: 0.039–3.554; q = 0.006), and *Roseburia* (PCR-positive: 0.000%, IQR: 0.000–0.003; PCR-negative: 0.109%, IQR: 0.000–1.637; q = 0.0006) (Figure 3C and D). Results remained consistent when excluding participants younger than 10 y old, with all major taxa retaining significance and similar effect sizes.

##### *Campylobacter* PCR-positive status is not associated with increased overall antimicrobial resistance gene load or antibiotic class diversity in the gut resistome.

**Figure 4.**
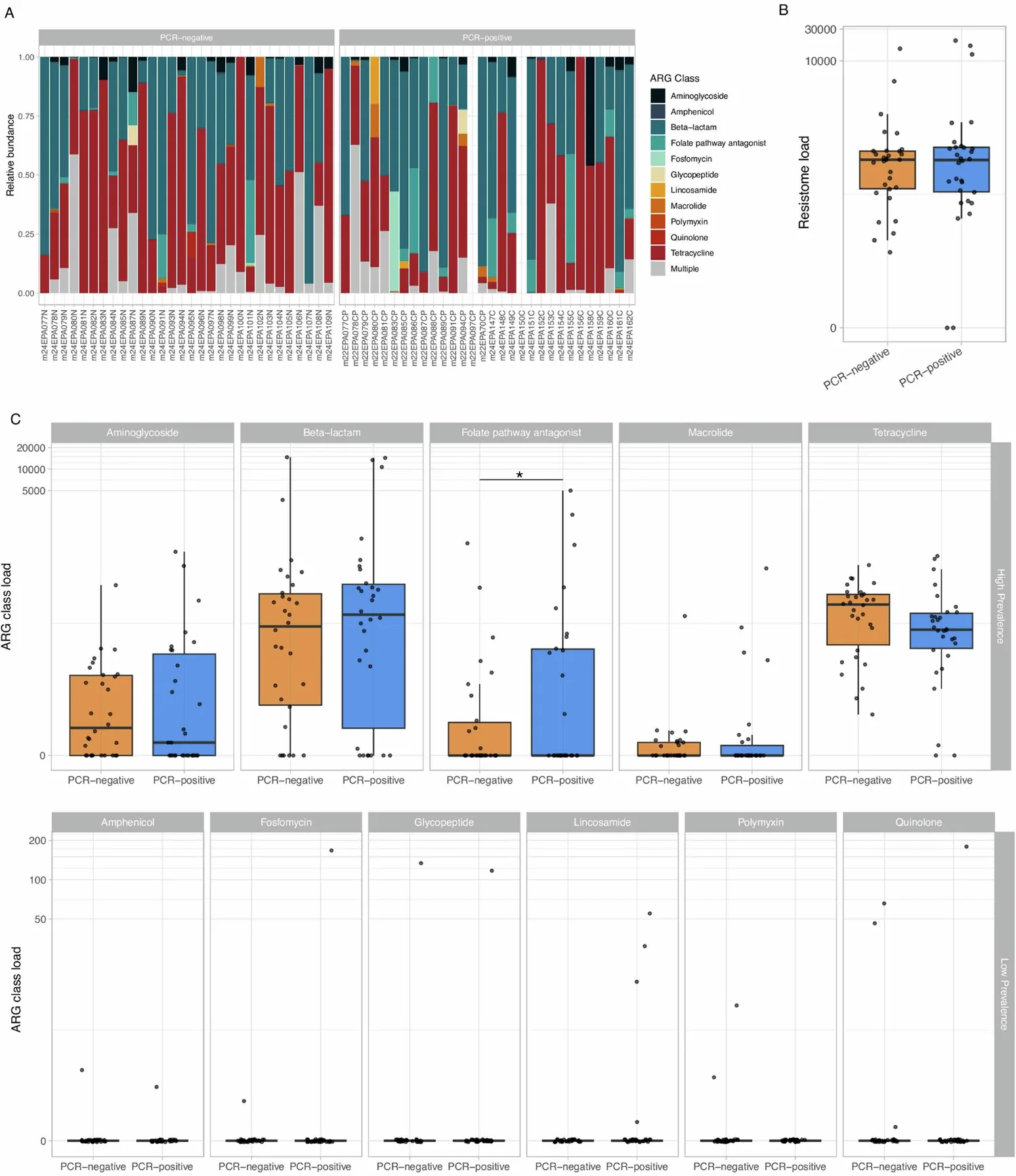
Resistome composition in PCR-positive and PCR-negative stool samples. (A) Relative abundance of antimicrobial resistance genes (ARGs). The stacked bar plot illustrates the proportional abundance of different ARG classes in each sample, calculated using reads per kilobase (RPK) values. Samples are grouped by *Campylobacter* PCR status (positive/negative). ARGs conferring resistance to multiple drug classes are categorized as “Multiple”. (B) Comparison of antimicrobial resistance gene (ARG) load between PCR-positive and PCR-negative samples. ARG abundance was calculated as reads per kilobase (RPK) normalized by total sequencing depth and expressed as ARG copies per million reads. Box plots show the median (center line), interquartile range (box), and 1.5 times the interquartile range (whiskers). Y-axis is shown on a pseudo-log10 scale, which allows visualization of values spanning several orders of magnitude while accommodating zero values. No significant difference was observed between groups (linear model, *P* = 0.79). (C) Comparison of antimicrobial resistance gene class load between PCR-positive and PCR-negative samples. Box plots show the load (ARG copies per million reads) of the five ARG classes with a prevalence above 5%: aminoglycoside, beta-lactam, folate pathway antagonist, macrolide, and tetracycline, and the load of ARG classes with a low prevalence. Box plots display the median (center line), interquartile range (box), and 1.5 times the interquartile range (whiskers). Y-axis is shown on a pseudo-log10 scale. Class-specific loads for high-prevalence ARG were compared using linear models adjusting for age and sequencing depth, and significance levels are reported as follows: *** *P* < 0.001, ** *P* < 0.01, * *P* < 0.05.

We investigated potential differences in resistome load and composition between PCR groups by profiling the resistomes against the ResFinder database, adjusting for age and sequencing run. A total of 331 ARGs were identified, with two *Campylobacter* PCR-positive samples showing no detectable antibiotic resistance genes (Figure 4A). *Campylobacter* PCR status was not associated with overall ARG load (linear model, *P* = 0.79) (Figure 4B). At the individual gene level, four tetracycline resistance genes were significantly lower in the PCR-positive group: *tet*(W) variants (q = 0.076) and *tet*(O) variants (q = 0.076–0.082). Analysis by antibiotic class revealed that folate pathway antagonist resistance genes were significantly higher in PCR-positive samples (*P* = 0.031), whereas no significant differences were observed for other major classes: aminoglycoside (*P* = 0.16), beta-lactam (*P* = 0.57), macrolide (*P* = 0.76), or tetracycline (*P* = 0.41) (Figure 4C). Results remained consistent when excluding participants < 10 y, with folate pathway antagonist genes maintaining significance (*P* = 0.018) and aminoglycoside genes showing borderline significance (*P* = 0.058).

To assess concordance between resistance determinants identified in cultured *Campylobacter* isolates and those detected in the corresponding stool metagenomes, we focused on horizontally transferable ARGs only. Chromosomal mutations (*gyrA_T86I*, *gyrA_P104S*, and *50S_L22_A103V*) were excluded as they are not detectable by read-based resistome profiling. Among the remaining assessable genes, concordance was partial: *tet*(O) was detected in the corresponding stool metagenome in 4 of the 9 samples where it was identified in the *Campylobacter* isolate, while *bla*_OXA-193_ was metagenomically detected in 2 of the 13 corresponding samples. *bla*_OXA-61_ was concordant in the single sample where it was identified in an isolate (1/1). At the cohort level, *tet*(O) was detected in 12 of 30 PCR-positive metagenomes and 22 of 30 PCR-negative metagenomes, consistent with its widespread distribution among commensal taxa rather than being *Campylobacter*-specific. By contrast, *bla*_OXA-193_ was detected in 3 of 30 PCR-positive metagenomes and none of the PCR-negative metagenomes, reflecting its association with *Campylobacter*.

##### *Campylobacter* relative abundance is highly variable and correlates with overall microbiome composition but not richness.

**Figure 5.**
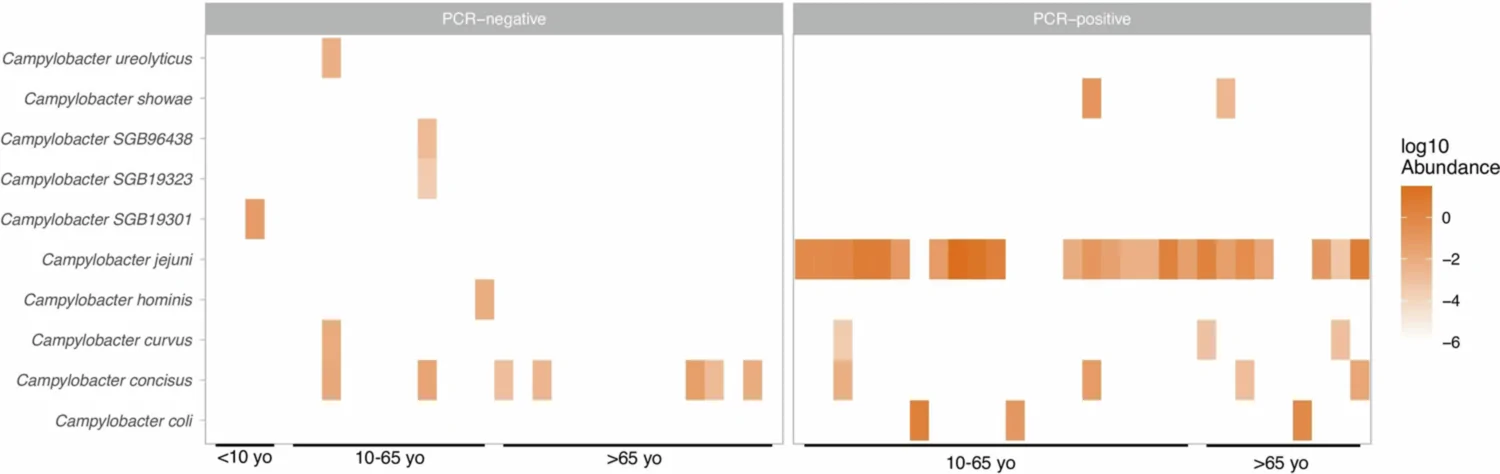
Distribution of *Campylobacter* species across PCR-positive and PCR-negative samples. The heatmap shows the relative abundance (log10-transformed) of individual *Campylobacter* species detected by WGS sequencing.

*Campylobacter* was detected in the metagenomic profiles of 27 of 30 PCR-positive stool samples, with relative abundances ranging from 0.1% to 31% of the microbial community (Figure 5). Culture recovered isolates from 20 of these samples (Table S2). Most culture-positive samples also showed detectable *Campylobacter* signal in the corresponding metagenomic profiles, whereas several PCR-positive samples without successful culture recovery showed low or undetectable *Campylobacter* abundance in the metagenomic data.

Three PCR-positive samples did not show detectable *Campylobacter* signal in the metagenomes: 24EPA147C (culture-negative), 24EPA151C (culture-negative), and 24EPA152C (culture-positive). In the latter sample (24EPA152C), *Campylobacter* was recovered by culture despite the absence of a detectable signal in the metagenomic profile.

Among the PCR-positive samples, *C. jejuni* and *C. coli* were the most abundant and prevalent species, with no observable age-related patterns. *Campylobacter* was also identified in 9 out of 30 PCR-negative samples, with relative abundances ranging from 0.1% to 5%. None of these PCR-negative samples contained detectable levels of *C. jejuni* or *C. coli*. One sample contained *C. ureolyticus* and another *C. curvus*. Additionally, some PCR-negative samples carried *Campylobacter* species commonly found in healthy individuals (*C. hominis*) or uncultured *Campylobacter* species (SGB96438, SGB19323, and SGB19301). The *Campylobacter* relative abundance in the PCR-positive samples was highly variable (median = 0.2, IQR: 0.023–1.872) and followed a very highly skewed distribution. Interestingly, the *Campylobacter* relative abundance was not significantly associated with the microbiome observed richness (Spearman correlation, *ρ* = −0.037, *P* = 0.84; linear model adjusting for age and sequencing depth, *P* = 0.14) but was associated with the global microbiome composition in the PCR-positive group (PERMANOVA on PCR-positive samples, 999 permutations, *P* = 0.015, R² = 6%).

## Discussion

In our study, *Campylobacter* -associated gastroenteritis stool samples demonstrated reduced diversity and lower species richness within the stool microbiome compared with symptomatic PCR-negative controls. Consistent with previous reports describing diversity loss during acute enteric infections, *Campylobacter*-positive gastroenteritis was associated with substantial alterations in gut microbial community composition.^35-39^ This shift included reduced abundance of commensal taxa such as *Rikenellaceae* and *Eubacteriaceae* and depletion of key short-chain fatty acid–producing genera, including *Blautia*, *Roseburia*, and *Alistipes*, which contribute to colonization resistance, gut barrier integrity, and immune regulation.^40^ At the same time, increased relative abundance of taxa including *Enterobacteriaceae* and *Campylobacteraceae* was observed, which is an abundance profile reported in inflammatory gut environments.^41^ Together, these alterations are consistent with reduced microbial resilience during *Campylobacter*-associated gastrointestinal disease, a pattern also described in both human and animal studies of enteric infections.^2^ ^,^ ^42-45^ Expansion of such opportunistic taxa is widely recognized as a hallmark of disrupted ecological stability within the gut during gastrointestinal infection and recovery.^12^

In our study, age emerged as a significant determinant of gut microbial composition, in line with well-documented age-dependent shifts in microbiome structure across the human lifespan.^46^ ^,^ ^47^ By contrast, gender and Bristol stool scores did not significantly influence microbiome diversity or beta-diversity, supporting the specificity of *Campylobacter*-associated changes and the robustness of our findings. This is consistent with previous observations showing that age exerts a stronger influence on gut microbial architecture than sex or stool form in many settings.^48^ However, it is important to note that variation in disease severity was not assessed in our study.

Notably, *Campylobacter* population abundance correlated with overall microbiome composition diversity but not with species richness. This is not contradictory to the group-level finding of reduced richness in PCR-positive samples; rather, it indicates that once infection is present, additional increases in *Campylobacter* abundance do not further reduce richness. Instead, the variation in pathogen load appears to drive which taxa shift, not how many taxa remain. This pattern is consistent with murine models showing pathogen-driven shifts in community structure without proportional loss of richness, supporting the idea that *Campylobacter* imposes selective rather than broad suppressive effects on the microbiome.^45^ Moreover, prior work demonstrates that gut inflammation leads to targeted loss of obligate anaerobes and expansion of pathobionts such as *Escherichia* and *Streptococcus,* ^49^ changes that mirror inflammation-associated dysbiosis driven by shifts in oxygen availability that favor facultative anaerobes like *Enterobacteriaceae*.^50^

While a subset of *Campylobacter* PCR-negative samples yielded low-level *Campylobacter* reads in metagenomic data, these findings should be interpreted cautiously. Such detections may reflect low-level carriage, transient presence, or taxa of uncertain clinical relevance rather than an active infection. Conversely, some PCR-positive samples did not show detectable *Campylobacter* signal in the metagenomic profiles, highlighting that metagenomic detection is also constrained by factors such as sequencing depth, relative abundance, and taxonomic classification sensitivity.^51^ Excluding or including these samples did not alter the overall differences in microbiota composition between PCR-positive and PCR-negative groups, supporting the robustness of our findings when using routine diagnostic PCR results as the case definition.^52^

Despite these taxonomic changes, interpreting resistome findings in this study requires an important caveat: detailed metadata on recent antibiotic exposure were not available, as samples were obtained as anonymized residual diagnostic material. Antibiotic exposure is a major determinant of both microbiome composition and resistome dynamics, and it is therefore not possible to exclude the possibility that some of the observed resistome variation reflects unmeasured prior antimicrobial exposure rather than infection status alone.^53^ Within these constraints, the absence of broad resistome expansion in PCR-positive samples remains an informative finding, contrasting with reports of significant increases in ARG diversity during acute infection with other enteric pathogens such as *Salmonella* and *Shigella.* ^13^ The only consistent differences were a small increase in folate pathway antagonist resistance genes and lower abundance of several *tet* (W) and *tet* (O) variants in PCR-positive samples. Overall, these results suggest that resistome alterations are limited in scope and are not a dominant feature of *Campylobacter*-associated disruption of the stool microbiome. One possible explanation for the lower abundance of tetracycline resistance determinants in PCR-positive *Campylobacter-containing* samples is that infection-associated ecological shifts may reduce the relative abundance of commensal taxa that commonly harbor these genes, while inflammation-associated taxa expand.

Our findings illustrate the expanded taxonomic resolution of shotgun metagenomics compared with standard PCR or culture. In our dataset, metagenomic profiling identified *C. ureolyticus*, *C. curvus*, and other uncultured *Campylobacter* species in several PCR-negative samples that were not captured by conventional assays. However, these detections should not be interpreted as direct evidence of superior clinical diagnostic sensitivity, as they may reflect low-level carriage, transient presence, or organisms of uncertain clinical significance rather than active infection. Accordingly, we view metagenomics as a complementary tool for pathogen detection and microbiome characterization rather than a replacement for established rapid clinical diagnostics.^51^ ^,^ ^54^ However, these advantages must be weighed against the current practical limitations of metagenomic implementation in clinical settings. Shotgun metagenomic sequencing remains relatively expensive and resource-intensive compared to targeted PCR or culture-based diagnostics, requiring specialized laboratory infrastructure and bioinformatics expertise that are not widely accessible in most clinical microbiology laboratories.^51^

In our study, dual-ST infections were observed in two cases, indicating polyclonal infections, a phenomenon increasingly recognized in both poultry and human infections.^55^ ^,^ ^56^

AMR profiling of the *Campylobacter* isolates revealed widespread carriage of *bla*_*OXA-193*_, *tet*(O), and *gyrA_T86I*, consistent with resistance to *β*-lactams and tetracyclines, a pattern frequently reported in both clinical and animal-derived *Campylobacter* strains.^57^ Notably, concordance between isolate-derived ARGs and metagenomic detection was partial and gene-dependent, likely reflecting the fact that genes such as *tet*(O) are widely distributed among commensal gut taxa and their metagenomic signal cannot be attributed exclusively to *Campylobacter*, whereas more *Campylobacter*-associated genes, such as *bla*_OXA-193_ showed a more restricted metagenomic distribution.^15^ ^,^ ^58^. This observation aligns with prior studies showing that stool resistomes often reflect a combination of commensal, environmental, and pathogenic sources, particularly in polymicrobial communities.^59^ Nevertheless, the detection of overlapping ARGs in both isolate genomes and metagenomes supports the utility of metagenomics in identifying resistance threats even when culture is not feasible, while also highlighting the need for complementary genomic validation to confirm taxonomic origin.

## Limitations

Our study provides relevant novel insights into *Campylobacter*-associated alterations in the gut microbiome and the resistome. As with any study, it is important to acknowledge the factors that may influence the generalizability and interpretation of the findings. First, the clinical cohort in this study was heterogeneous in age and sex, potentially introducing confounders that could mask pathogen-specific microbiome or ARG signatures. Second, the PCR-negative comparator group was comprised of samples from individuals with symptomatic diarrhea with no known enteric pathogen found using a rapid gastroenteric testing panel. The samples tested negative for *Salmonella*, *Shigella*, shiga toxin-producing *Escherichia coli*, *Campylobacter*, *Cryptosporidium*, and *Giardia*; however, viral gastroenteritis, infections caused by pathogens not included on the panel, low-level infections below PCR thresholds, or noninfectious gastrointestinal conditions cannot be ruled out. Such heterogeneity may reduce the specificity of between-group comparisons. Third, the limited clinical and epidemiological metadata available, such as recent antibiotic use, travel history, diet, or underlying gastrointestinal disorders, prevented adjustment for these potentially influential variables. Antibiotic exposure is a determinant of both gut microbiome and functional resistome characteristics.^49^ ^,^ ^53^ Risk factor associations between antibiotic exposure and resistome characteristics were beyond the scope of this study.

Finally, our cross-sectional design precludes temporal inference; therefore, pre-existing microbiome alterations (e.g., reduced low diversity, expansion of inflammation-associated taxa) cannot be excluded as associated risk factors for *Campylobacter* colonization rather than the infection itself being associated with the resulting dysbiosis. Longitudinal studies with pre- and postinfection sampling are required to further explain changes in microbiome ecology after *Campylobacter* infection.

## Conclusion

Our findings demonstrate that *Campylobacter*-positive gastroenteritis is associated with a distinct pattern of gut microbiome disruption characterized by reduced richness and shifts in key taxonomic groups, without a corresponding broad expansion of the gut resistome. The consistency of the observed microbiome signals supports the relevance of these findings and can be expanded on through longitudinal studies to clarify the temporal dynamics, specificity, and clinical management implications of *Campylobacter*-associated microbiome alterations.

## Supplementary Material

Supplementary Material Supplementary_materials_Reviewed_BD__1_.docx

## Data Availability

All supporting data, code, and protocols are included in the article or are available as supplementary data files. The online version of this article contains two supplementary figures (Figures S1–S4) and four supplementary tables (Tables S1–S3). All sequenced *Campylobacter jejuni* isolate data are available in the National Center for Biotechnology Information (NCBI) Sequence Read Archive under the Bioproject accession numbers PRJNA1346933 and PRJNA1046283 [https://dataview.ncbi.nlm.nih.gov/object/PRJNA1346933?reviewer=3p6n4u0qkfkkg42vsng97q4l9s]. Sequence read archive (SRA) accession numbers and associated metadata are provided in the supplementary material of this study (Table S3).

## Impact statement

This study characterizes gut microbiome alterations associated with *Campylobacter* infection, revealing distinct taxonomic shifts marked by reduced richness and increased abundance of inflammation-associated taxa. By combining culture-based isolate genomics with shotgun metagenomic profiling, we show that these microbial disruptions occur without a corresponding increase in the burden of antimicrobial resistance genes (ARG), contrasting with reports of broader resistome expansion in other enteric infections. Our findings also show that metagenomics can detect low-abundance *Campylobacter* species that may be missed by routine PCR or culture, highlighting its potential as a complementary tool for microbiome characterization as well as pathogen detection.

## Data Summary

All relevant supporting data are available in supplementary data files. The online version of this article contains three supplementary tables and four supplementary figures.

All *Campylobacter* isolate genomes and stool metagenome sequences are available in the National Center for Biotechnology Information (NCBI) Sequence Read Archive (SRA) under the Bioproject accession numbers PRJNA1346933 and PRJNA1046283 [https://dataview.ncbi.nlm.nih.gov/object/PRJNA1346933?reviewer=3p6n4u0qkfkkg42vsng97q4l9s]. SRA accession numbers and associated metadata for isolate genomes and metagenomes are included in the supplementary table S3. All codes and parameters of all computational tools and analysis steps, as well as RStudio codes, were deposited on GitHub [https://github.com/aponsero/QIB_WGScleanup].

## Abbreviations

%: percent
°C: Degree Celsius
AMR: antimicrobial resistance
*bla*_OXA_: beta-lactamase oxacillinase gene
*C. jejuni*: *Campylobacter jejuni*
CBA: Columbia blood agar
CDSs: Coding-DNA sequences
DNA: deoxyribonucleic acid
EPA: Eastern Pathology Laboratory
*gyrA*: DNA gyrase subunit A
HTA: Health Technology Assessment
ISO: International Organization for Standardization
mCCDA: modified Charcoal Cefoperazone Deoxycholate Agar
ml: milliliter
MLST: Multilocus sequence typing
PBS: Phosphate-buffered saline
PCR: polymerase chain reaction
ONT: Oxford Nanopore Technologies
RNA: ribonuclease acid
Spp.: species
ST: sequence type
*Tet*: tetracycline gene
UK: United Kingdom
V: Version
WGS: whole genome sequencing
*β*-lactam: Beta lactam
μl: microlitre
μm: micrometer
