# Supplementary material for: Gut microbiome dysbiosis and functional alterations in Campylobacter-associated gastroenteritis using metagenomic approaches
Source: Gut Microbes Rep. 2026 Aug 18;3(1):2688065. doi: 10.1080/29933935.2026.2688065 (PMC13488468; doi:10.1080/29933935.2026.2688065)
Supplement: Supplementary file 1 [file KGMR_A_2688065_SM8939.docx]

**Supplementary materials**

**Table S1.** Metadata and sample information for *Campylobacter* PCR-positive and PCR-negative stool samples.

| **Sample ID** | **BioProject** | **SRA accession number** | **PCR** | **Bristol Scale** | **Culture** | **DD/MM/YY of collection** | **Gender** | **DD/MM/YY of birth** |
| --- | --- | --- | --- | --- | --- | --- | --- | --- |
| 22EPA070CP | PRJNA1046283 | SRR27129239 | Ca+ | 5 | C- | 01/05/2022 | M | 19/07/1982 |
| 22EPA077CP | PRJNA1046283 | SRR27129238 | Ca+ | 6 | C+ | 01/06/2022 | M | 09/01/1992 |
| 22EPA078CP | PRJNA1046283 | SRR27129237 | Ca+ | 7 | C+ | 01/06/2022 | M | 19/10/1930 |
| 22EPA079CP | PRJNA1046283 | SRR27129236 | Ca+ | 5 | C+ | 01/06/2022 | M | 01/09/1996 |
| 22EPA080CP | PRJNA1046283 | SRR27129235 | Ca+ | 6 | C+ | 01/06/2022 | M | 22/02/1960 |
| 22EPA081CP | PRJNA1046283 | SRR27129233 | Ca+ | 7 | C+ | 01/06/2022 | M | 12/01/1955 |
| 22EPA083CP | PRJNA1046283 | SRR27129232 | Ca+ | 6 | C+ | 01/06/2022 | F | 15/11/1994 |
| 22EPA085CP | PRJNA1046283 | SRR27129231 | Ca+ | 7 | C+ | 01/07/2022 | M | 24/06/2002 |
| 22EPA086CP | PRJNA1046283 | SRR27129230 | Ca+ | 6 | C+ | 01/06/2022 | F | 24/09/1998 |
| 22EPA087CP | PRJNA1046283 | SRR27129229 | Ca+ | 7 | C+ | 01/07/2022 | M | 12/09/1961 |
| 22EPA088CP | PRJNA1046283 | SRR27129228 | Ca+ | 7 | C+ | 01/07/2022 | F | 22/12/1953 |
| 22EPA089CP | PRJNA1046283 | SRR27129227 | Ca+ | 7 | C- | 01/07/2022 | M | 15/11/1943 |
| 22EPA091CP | PRJNA1046283 | SRR27129226 | Ca+ | 6 | C- | 01/06/2022 | M | 03/04/1978 |
| 22EPA094CP | PRJNA1046283 | SRR27129225 | Ca+ | 7 | C- | 01/07/2022 | F | 02/09/1963 |
| 22EPA097CP | PRJNA1046283 | SRR27129224 | Ca+ | 7 | C- | 01/07/2022 | M | 28/07/1998 |
| 24EPA077N | PRJNA1346933 | SRR35848378 | Ca- | 7 | C- | 01/07/2024 | M | 31/08/1951 |
| 24EPA078N | PRJNA1346933 | SRR35848377 | Ca- | 6 | C- | 01/07/2024 | F | 28/03/1952 |
| 24EPA079N | PRJNA1346933 | SRR35848352 | Ca- | 6 | C- | 01/07/2024 | F | 22/02/1938 |
| 24EPA080N | PRJNA1346933 | SRR35848341 | Ca- | 7 | C- | 01/07/2024 | M | 15/04/1953 |
| 24EPA081N | PRJNA1346933 | SRR35848372 | Ca- | 6 | C- | 01/07/2024 | F | 07/03/1996 |
| 24EPA082N | PRJNA1346933 | SRR35848366 | Ca- | 7 | C- | 01/07/2024 | F | 24/06/1973 |
| 24EPA083N | PRJNA1346933 | SRR35848365 | Ca- | 6 | C- | 01/07/2024 | M | 25/04/2024 |
| 24EPA084N | PRJNA1346933 | SRR35848364 | Ca- | 6 | C- | 01/07/2024 | F | 15/12/2016 |
| 24EPA085N | PRJNA1346933 | SRR35848363 | Ca- | 7 | C- | 01/07/2024 | F | 24/01/1947 |
| 24EPA087N | PRJNA1346933 | SRR35848361 | Ca- | 7 | C- | 01/07/2024 | F | 07/02/2019 |
| 24EPA089N | PRJNA1346933 | SRR35848376 | Ca- | 7 | C- | 01/07/2024 | F | 16/08/1941 |
| 24EPA090N | PRJNA1346933 | SRR35848362 | Ca- | 7 | C- | 01/07/2024 | F | 07/03/1948 |
| 24EPA091N | PRJNA1346933 | SRR35848360 | Ca- | 7 | C- | 01/07/2024 | M | 03/01/1984 |
| 24EPA093N | PRJNA1346933 | SRR35848359 | Ca- | 7 | C- | 01/07/2024 | F | 05/04/1975 |
| 24EPA094N | PRJNA1346933 | SRR35848358 | Ca- | 6 | C- | 01/08/2024 | M | 02/08/1955 |
| 24EPA095N | PRJNA1346933 | SRR35848357 | Ca- | 6 | C- | 01/08/2024 | M | 14/07/1956 |
| 24EPA096N | PRJNA1346933 | SRR35848356 | Ca- | 6 | C- | 01/08/2024 | F | 24/10/1940 |
| 24EPA097N | PRJNA1346933 | SRR35848355 | Ca- | 5 | C- | 01/08/2024 | F | 26/08/1961 |
| 24EPA098N | PRJNA1346933 | SRR35848354 | Ca- | 7 | C- | 01/08/2024 | F | 27/06/1959 |
| 24EPA099N | PRJNA1346933 | SRR35848353 | Ca- | 7 | C- | 01/08/2024 | M | 08/05/1950 |
| 24EPA100N | PRJNA1346933 | SRR35848351 | Ca- | 7 | C- | 01/08/2024 | M | 10/11/1947 |
| 24EPA101N | PRJNA1346933 | SRR35848350 | Ca- | 7 | C- | 01/08/2024 | F | 08/04/1969 |
| 24EPA102N | PRJNA1346933 | SRR35848349 | Ca- | 7 | C- | 01/08/2024 | F | 02/07/2024 |
| 24EPA103N | PRJNA1346933 | SRR35848348 | Ca- | 7 | C- | 01/08/2024 | M | 16/01/1964 |
| 24EPA104N | PRJNA1346933 | SRR35848347 | Ca- | 7 | C- | 01/08/2024 | F | 01/11/1977 |
| 24EPA105N | PRJNA1346933 | SRR35848346 | Ca- | 7 | C- | 01/08/2024 | M | 23/04/1957 |
| 24EPA106N | PRJNA1346933 | SRR35848345 | Ca- | 7 | C- | 01/08/2024 | F | 15/10/1947 |
| 24EPA107N | PRJNA1346933 | SRR35848344 | Ca- | 5 | C- | 01/09/2024 | F | 05/01/1956 |
| 24EPA108N | PRJNA1346933 | SRR35848343 | Ca- | 6 | C- | 01/09/2024 | F | 07/06/1965 |
| 24EPA109N | PRJNA1346933 | SRR35848342 | Ca- | 6 | C- | 01/09/2024 | M | 14/10/1993 |
| 24EPA147C | PRJNA1346933 | SRR35848340 | Ca+ | 6 | C- | 01/09/2024 | M | 26/09/1944 |
| 24EPA148C | PRJNA1346933 | SRR35848339 | Ca+ | 7 | C- | 01/09/2024 | M | 20/01/1984 |
| 24EPA149C | PRJNA1346933 | SRR35853138 | Ca+ | 6 | C+ | 01/09/2024 | M | 03/07/1955 |
| 24EPA150C | PRJNA1346933 | SRR35853243 | Ca+ | 7 | C+ | 01/09/2024 | M | 21/07/1981 |
| 24EPA151C | PRJNA1346933 | SRR35848336 | Ca+ | 6 | C- | 01/09/2024 | M | 26/09/1964 |
| 24EPA152C | PRJNA1346933 | SRR35853239 | Ca+ | 7 | C+ | 01/09/2024 | F | 13/01/1975 |
| 24EPA153C | PRJNA1346933 | SRR35853223 | Ca+ | 6 | C+ | 01/09/2024 | M | 21/03/1986 |
| 24EPA154C | PRJNA1346933 | SRR35853314 | Ca+ | 7 | C+ | 01/09/2024 | M | 26/05/1972 |
| 24EPA155C | PRJNA1346933 | SRR35853308 | Ca+ | 7 | C+ | 01/09/2024 | M | 01/09/1986 |
| 24EPA156C | PRJNA1346933 | SRR35853296 | Ca+ | 6 | C+ | 01/09/2024 | F | 08/02/1964 |
| 24EPA158C | PRJNA1346933 | SRR35853288 | Ca+ | 7 | C+ | 01/09/2024 | F | 02/11/1964 |
| 24EPA159C | PRJNA1346933 | SRR35853129 | Ca+ | 7 | C+ | 01/09/2024 | M | 16/01/1947 |
| 24EPA160C | PRJNA1346933 | SRR35848369 | Ca+ | 6 | C- | 01/09/2024 | F | 02/01/1945 |
| 24EPA161C | PRJNA1346933 | SRR35853122 | Ca+ | 7 | C+ | 01/09/2024 | F | 24/09/1946 |
| 24EPA162C | PRJNA1346933 | SRR35848367 | Ca+ | 7 | C- | 01/09/2024 | M | 13/03/1972 |

**Ca+/Ca- :** *Campylobacter* PCR positive/negative, **C+/C-:** *Campylobacter* culture positive/negative. **F/M:** Female/Male.

**Table S2.** Characteristics of *Campylobacter* isolates recovered from *Campylobacter* PCR-positive stool samples.

| **Sample ID** | **Year of isolation** | **Number of isolates** | **Species** | **ST (No. of isolates)** | **AMR determinant genes (No. of isolates)** |
| --- | --- | --- | --- | --- | --- |
| 22EPA087C | 2022 | 18 | *C. coli* | 1055 | *tet*(O) |
| 24EPA149C | 2024 | 3 | *C. coli* | 825 | - |
| 22EPA077C | 2022 | 10 | *C. jejuni* | 464 | (9): *bla*_OXA-193_ ; *tet*(O) ; *50S_L22_A103V* ; *gyr*A_T86 , (1): *bla*_OXA-193_ ; tet(O) ; *50S_L22_A103V* |
| 22EPA078C | 2022 | 19 | *C. jejuni* | 137 | *bla*_OXA-193_ |
| 22EPA079C | 2022 | 20 | *C. jejuni* | 50 | *bla*_OXA-193_ |
| 22EPA080C | 2022 | 19 | *C. jejuni* | 441 | *bla*_OXA-193_ ; *gyr*A_P104S ; *gyr*A_T86I |
| 22EPA081C | 2022 | 10 | *C. jejuni* | 677 | *bla*_OXA-447_ |
| 22EPA083C | 2022 | 20 | *C. jejuni* | 6532 | *bla*_OXA-193_ ; *tet*(O) ; *50S_L22_A103V* ; *gyr*A_T86 ; *aad*E |
| 22EPA085C | 2022 | 20 | *C. jejuni* | (11): 21, (9): 48 | (11): *bla*_OXA-193_, (9): *bla*_OXA-61_ |
| 22EPA086C | 2022 | 20 | *C. jejuni* | (9): 400, (11): 10719 | (9): *tet*(O) ; *50S_L22_A103V* ; *gyr*A_T86 , (11): *bla*_OXA-623_ |
| 22EPA088C | 2022 | 14 | *C. jejuni* | 447 | - |
| 24EPA150C | 2024 | 7 | *C. jejuni* | 6175 | *bla*_OXA-193_; *tet*(O) ; *gyr*A_T86 |
| 24EPA152C | 2024 | 10 | *C. jejuni* | 9897 | (8): *bla*_OXA-193_; *tet*(O) ; *gyr*A_T86I , (1): *tet*(O) ; *gyr*A_T86I |
| 24EPA153C | 2024 | 9 | *C. jejuni* | 45 | *bla*_OXA-193_ |
| 24EPA154C | 2024 | 9 | *C. jejuni* | 21 | *bla*_OXA-193_ |
| 24EPA155C | 2024 | 10 | *C. jejuni* | 19 | *bla*_OXA-193_; *50S_L22_A103V* ; *gyr*A_T86 |
| 24EPA156C | 2024 | 8 | *C. jejuni* | 50 | (8): *bla*_OXA-193_; *tet*(O) ; *gyr*A_T86I |
| 24EPA158C | 2024 | 3 | *C. jejuni* | 508 | - |
| 24EPA159C | 2024 | 6 | *C. jejuni* | 6175 | (8): *bla*_OXA-193_; *tet*(O) ; *gyr*A_T86I |
| 24EPA161C | 2024 | 8 | *C. jejuni* | 6532 | *tet*(O) ; *gyr*A_T86 ; *aad*E |

**C** : *Campylobacter*, **ST**: sequence type, **AMR**: antimicrobial resistance, **(n)**: number of isolates.

**Table S3.** Metadata and sample information for *Campylobacter* genomes recovered from stool samples.

| **Sample Name** | **Species** | **Method** | **Location** | **DD/MM/YY of collection** | **Bioproject** | **SRA accession number** |
| --- | --- | --- | --- | --- | --- | --- |
| PID-1108-22EPA078CP1 | *C. jejuni* | Cell culture | Norwich, UK | 01/06/2022 | PRJNA1346933 | SRR35853344 |
| PID-1108-22EPA078CP10 | *C. jejuni* | Cell culture | Norwich, UK | 01/06/2022 | PRJNA1346933 | SRR35853345 |
| PID-1108-22EPA078CP11 | *C. jejuni* | Cell culture | Norwich, UK | 01/06/2022 | PRJNA1346933 | SRR35853218 |
| PID-1108-22EPA078CP12 | *C. jejuni* | Cell culture | Norwich, UK | 01/06/2022 | PRJNA1346933 | SRR35853108 |
| PID-1108-22EPA078CP13 | *C. jejuni* | Cell culture | Norwich, UK | 01/06/2022 | PRJNA1346933 | SRR35853209 |
| PID-1108-22EPA078CP14 | *C. jejuni* | Cell culture | Norwich, UK | 01/06/2022 | PRJNA1346933 | SRR35853198 |
| PID-1108-22EPA078CP15 | *C. jejuni* | Cell culture | Norwich, UK | 01/06/2022 | PRJNA1346933 | SRR35853187 |
| PID-1108-22EPA078CP16 | *C. jejuni* | Cell culture | Norwich, UK | 01/06/2022 | PRJNA1346933 | SRR35853176 |
| PID-1108-22EPA078CP17 | *C. jejuni* | Cell culture | Norwich, UK | 01/06/2022 | PRJNA1346933 | SRR35853165 |
| PID-1108-22EPA078CP18 | *C. jejuni* | Cell culture | Norwich, UK | 01/06/2022 | PRJNA1346933 | SRR35853282 |
| PID-1108-22EPA078CP19 | *C. jejuni* | Cell culture | Norwich, UK | 01/06/2022 | PRJNA1346933 | SRR35853344 |
| PID-1108-22EPA078CP2 | *C. jejuni* | Cell culture | Norwich, UK | 01/06/2022 | PRJNA1346933 | SRR35853333 |
| PID-1108-22EPA078CP3 | *C. jejuni* | Cell culture | Norwich, UK | 01/06/2022 | PRJNA1346933 | SRR35853322 |
| PID-1108-22EPA078CP4 | *C. jejuni* | Cell culture | Norwich, UK | 01/06/2022 | PRJNA1346933 | SRR35853267 |
| PID-1108-22EPA078CP5 | *C. jejuni* | Cell culture | Norwich, UK | 01/06/2022 | PRJNA1346933 | SRR35853256 |
| PID-1108-22EPA078CP6 | *C. jejuni* | Cell culture | Norwich, UK | 01/06/2022 | PRJNA1346933 | SRR35853245 |
| PID-1108-22EPA078CP7 | *C. jejuni* | Cell culture | Norwich, UK | 01/06/2022 | PRJNA1346933 | SRR35853150 |
| PID-1108-22EPA078CP8 | *C. jejuni* | Cell culture | Norwich, UK | 01/06/2022 | PRJNA1346933 | SRR35853139 |
| PID-1108-22EPA078CP9 | *C. jejuni* | Cell culture | Norwich, UK | 01/06/2022 | PRJNA1346933 | SRR35853240 |
| PID-1108-22EPA079CP1 | *C. jejuni* | Cell culture | Norwich, UK | 01/06/2022 | PRJNA1346933 | SRR35853306 |
| PID-1108-22EPA079CP10 | *C. jejuni* | Cell culture | Norwich, UK | 01/06/2022 | PRJNA1346933 | SRR35853217 |
| PID-1108-22EPA079CP11 | *C. jejuni* | Cell culture | Norwich, UK | 01/06/2022 | PRJNA1346933 | SRR35853306 |
| PID-1108-22EPA079CP12 | *C. jejuni* | Cell culture | Norwich, UK | 01/06/2022 | PRJNA1346933 | SRR35853295 |
| PID-1108-22EPA079CP13 | *C. jejuni* | Cell culture | Norwich, UK | 01/06/2022 | PRJNA1346933 | SRR35853128 |
| PID-1108-22EPA079CP14 | *C. jejuni* | Cell culture | Norwich, UK | 01/06/2022 | PRJNA1346933 | SRR35853117 |
| PID-1108-22EPA079CP15 | *C. jejuni* | Cell culture | Norwich, UK | 01/06/2022 | PRJNA1346933 | SRR35853113 |
| PID-1108-22EPA079CP16 | *C. jejuni* | Cell culture | Norwich, UK | 01/06/2022 | PRJNA1346933 | SRR35853112 |
| PID-1108-22EPA079CP17 | *C. jejuni* | Cell culture | Norwich, UK | 01/06/2022 | PRJNA1346933 | SRR35853111 |
| PID-1108-22EPA079CP18 | *C. jejuni* | Cell culture | Norwich, UK | 01/06/2022 | PRJNA1346933 | SRR35853110 |
| PID-1108-22EPA079CP19 | *C. jejuni* | Cell culture | Norwich, UK | 01/06/2022 | PRJNA1346933 | SRR35853109 |
| PID-1108-22EPA079CP2 | *C. jejuni* | Cell culture | Norwich, UK | 01/06/2022 | PRJNA1346933 | SRR35853107 |
| PID-1108-22EPA079CP20 | *C. jejuni* | Cell culture | Norwich, UK | 01/06/2022 | PRJNA1346933 | SRR35853106 |
| PID-1108-22EPA079CP3 | *C. jejuni* | Cell culture | Norwich, UK | 01/06/2022 | PRJNA1346933 | SRR35853105 |
| PID-1108-22EPA079CP4 | *C. jejuni* | Cell culture | Norwich, UK | 01/06/2022 | PRJNA1346933 | SRR35853104 |
| PID-1108-22EPA079CP5 | *C. jejuni* | Cell culture | Norwich, UK | 01/06/2022 | PRJNA1346933 | SRR35853215 |
| PID-1108-22EPA079CP6 | *C. jejuni* | Cell culture | Norwich, UK | 01/06/2022 | PRJNA1346933 | SRR35853214 |
| PID-1108-22EPA079CP7 | *C. jejuni* | Cell culture | Norwich, UK | 01/06/2022 | PRJNA1346933 | SRR35853213 |
| PID-1108-22EPA079CP8 | *C. jejuni* | Cell culture | Norwich, UK | 01/06/2022 | PRJNA1346933 | SRR35853212 |
| PID-1108-22EPA079CP9 | *C. jejuni* | Cell culture | Norwich, UK | 01/06/2022 | PRJNA1346933 | SRR35853211 |
| PID-1108-22EPA081CP1 | *C. jejuni* | Cell culture | Norwich, UK | 01/06/2022 | PRJNA1346933 | SRR35853210 |
| PID-1108-22EPA081CP10 | *C. jejuni* | Cell culture | Norwich, UK | 01/06/2022 | PRJNA1346933 | SRR35853208 |
| PID-1108-22EPA081CP2 | *C. jejuni* | Cell culture | Norwich, UK | 01/06/2022 | PRJNA1346933 | SRR35853207 |
| PID-1108-22EPA081CP3 | *C. jejuni* | Cell culture | Norwich, UK | 01/06/2022 | PRJNA1346933 | SRR35853206 |
| PID-1108-22EPA081CP4 | *C. jejuni* | Cell culture | Norwich, UK | 01/06/2022 | PRJNA1346933 | SRR35853205 |
| PID-1108-22EPA081CP5 | *C. jejuni* | Cell culture | Norwich, UK | 01/06/2022 | PRJNA1346933 | SRR35853204 |
| PID-1108-22EPA081CP6 | *C. jejuni* | Cell culture | Norwich, UK | 01/06/2022 | PRJNA1346933 | SRR35853203 |
| PID-1108-22EPA081CP7 | *C. jejuni* | Cell culture | Norwich, UK | 01/06/2022 | PRJNA1346933 | SRR35853202 |
| PID-1108-22EPA081CP8 | *C. jejuni* | Cell culture | Norwich, UK | 01/06/2022 | PRJNA1346933 | SRR35853201 |
| PID-1108-22EPA081CP9 | *C. jejuni* | Cell culture | Norwich, UK | 01/06/2022 | PRJNA1346933 | SRR35853200 |
| PID-1150-22EPA085CP1 | *C. jejuni* | Cell culture | Norwich, UK | 01/07/2024 | PRJNA1346933 | SRR35853195 |
| PID-1150-22EPA085CP10 | *C. jejuni* | Cell culture | Norwich, UK | 01/07/2024 | PRJNA1346933 | SRR35853197 |
| PID-1150-22EPA085CP11 | *C. jejuni* | Cell culture | Norwich, UK | 01/07/2024 | PRJNA1346933 | SRR35853196 |
| PID-1150-22EPA085CP12 | *C. jejuni* | Cell culture | Norwich, UK | 01/07/2024 | PRJNA1346933 | SRR35853195 |
| PID-1150-22EPA085CP13 | *C. jejuni* | Cell culture | Norwich, UK | 01/07/2024 | PRJNA1346933 | SRR35853194 |
| PID-1150-22EPA085CP14 | *C. jejuni* | Cell culture | Norwich, UK | 01/07/2024 | PRJNA1346933 | SRR35853193 |
| PID-1150-22EPA085CP15 | *C. jejuni* | Cell culture | Norwich, UK | 01/07/2024 | PRJNA1346933 | SRR35853192 |
| PID-1150-22EPA085CP16 | *C. jejuni* | Cell culture | Norwich, UK | 01/07/2024 | PRJNA1346933 | SRR35853191 |
| PID-1150-22EPA085CP17 | *C. jejuni* | Cell culture | Norwich, UK | 01/07/2024 | PRJNA1346933 | SRR35853190 |
| PID-1150-22EPA085CP18 | *C. jejuni* | Cell culture | Norwich, UK | 01/07/2024 | PRJNA1346933 | SRR35853189 |
| PID-1150-22EPA085CP19 | *C. jejuni* | Cell culture | Norwich, UK | 01/07/2024 | PRJNA1346933 | SRR35853188 |
| PID-1150-22EPA085CP2 | *C. jejuni* | Cell culture | Norwich, UK | 01/07/2024 | PRJNA1346933 | SRR35853185 |
| PID-1150-22EPA085CP20 | *C. jejuni* | Cell culture | Norwich, UK | 01/07/2024 | PRJNA1346933 | SRR35853185 |
| PID-1150-22EPA085CP3 | *C. jejuni* | Cell culture | Norwich, UK | 01/07/2024 | PRJNA1346933 | SRR35853184 |
| PID-1150-22EPA085CP4 | *C. jejuni* | Cell culture | Norwich, UK | 01/07/2024 | PRJNA1346933 | SRR35853183 |
| PID-1150-22EPA085CP5 | *C. jejuni* | Cell culture | Norwich, UK | 01/07/2024 | PRJNA1346933 | SRR35853182 |
| PID-1150-22EPA085CP6 | *C. jejuni* | Cell culture | Norwich, UK | 01/07/2024 | PRJNA1346933 | SRR35853181 |
| PID-1150-22EPA085CP7 | *C. jejuni* | Cell culture | Norwich, UK | 01/07/2024 | PRJNA1346933 | SRR35853180 |
| PID-1150-22EPA085CP8 | *C. jejuni* | Cell culture | Norwich, UK | 01/07/2024 | PRJNA1346933 | SRR35853179 |
| PID-1150-22EPA085CP9 | *C. jejuni* | Cell culture | Norwich, UK | 01/06/2022 | PRJNA1346933 | SRR35853178 |
| PID-1150-22EPA086CP1 | *C. jejuni* | Cell culture | Norwich, UK | 01/06/2022 | PRJNA1346933 | SRR35853177 |
| PID-1150-22EPA086CP10 | *C. jejuni* | Cell culture | Norwich, UK | 01/06/2022 | PRJNA1346933 | SRR35853175 |
| PID-1150-22EPA086CP11 | *C. jejuni* | Cell culture | Norwich, UK | 01/06/2022 | PRJNA1346933 | SRR35853174 |
| PID-1150-22EPA086CP12 | *C. jejuni* | Cell culture | Norwich, UK | 01/06/2022 | PRJNA1346933 | SRR35853173 |
| PID-1150-22EPA086CP13 | *C. jejuni* | Cell culture | Norwich, UK | 01/06/2022 | PRJNA1346933 | SRR35853172 |
| PID-1150-22EPA086CP14 | *C. jejuni* | Cell culture | Norwich, UK | 01/06/2022 | PRJNA1346933 | SRR35853171 |
| PID-1150-22EPA086CP15 | *C. jejuni* | Cell culture | Norwich, UK | 01/06/2022 | PRJNA1346933 | SRR35853170 |
| PID-1150-22EPA086CP16 | *C. jejuni* | Cell culture | Norwich, UK | 01/06/2022 | PRJNA1346933 | SRR35853169 |
| PID-1150-22EPA086CP17 | *C. jejuni* | Cell culture | Norwich, UK | 01/06/2022 | PRJNA1346933 | SRR35853168 |
| PID-1150-22EPA086CP18 | *C. jejuni* | Cell culture | Norwich, UK | 01/06/2022 | PRJNA1346933 | SRR35853167 |
| PID-1150-22EPA086CP19 | *C. jejuni* | Cell culture | Norwich, UK | 01/06/2022 | PRJNA1346933 | SRR35853166 |
| PID-1150-22EPA086CP2 | *C. jejuni* | Cell culture | Norwich, UK | 01/06/2022 | PRJNA1346933 | SRR35853164 |
| PID-1150-22EPA086CP20 | *C. jejuni* | Cell culture | Norwich, UK | 01/06/2022 | PRJNA1346933 | SRR35853163 |
| PID-1150-22EPA086CP3 | *C. jejuni* | Cell culture | Norwich, UK | 01/06/2022 | PRJNA1346933 | SRR35853162 |
| PID-1150-22EPA086CP4 | *C. jejuni* | Cell culture | Norwich, UK | 01/06/2022 | PRJNA1346933 | SRR35853161 |
| PID-1150-22EPA086CP5 | *C. jejuni* | Cell culture | Norwich, UK | 01/06/2022 | PRJNA1346933 | SRR35853160 |
| PID-1150-22EPA086CP6 | *C. jejuni* | Cell culture | Norwich, UK | 01/06/2022 | PRJNA1346933 | SRR35853287 |
| PID-1150-22EPA086CP7 | *C. jejuni* | Cell culture | Norwich, UK | 01/06/2022 | PRJNA1346933 | SRR35853286 |
| PID-1150-22EPA086CP8 | *C. jejuni* | Cell culture | Norwich, UK | 01/06/2022 | PRJNA1346933 | SRR35853285 |
| PID-1150-22EPA086CP9 | *C. jejuni* | Cell culture | Norwich, UK | 01/06/2022 | PRJNA1346933 | SRR35853284 |
| PID-1150-22EPA087CP1 | *C. coli* | Cell culture | Norwich, UK | 01/07/2024 | PRJNA1346933 | SRR35853281 |
| PID-1150-22EPA087CP10 | *C. coli* | Cell culture | Norwich, UK | 01/07/2024 | PRJNA1346933 | SRR35853281 |
| PID-1150-22EPA087CP11 | *C. coli* | Cell culture | Norwich, UK | 01/07/2024 | PRJNA1346933 | SRR35853280 |
| PID-1150-22EPA087CP12 | *C. coli* | Cell culture | Norwich, UK | 01/07/2024 | PRJNA1346933 | SRR35853279 |
| PID-1150-22EPA087CP13 | *C. coli* | Cell culture | Norwich, UK | 01/07/2024 | PRJNA1346933 | SRR35853278 |
| PID-1150-22EPA087CP14 | *C. coli* | Cell culture | Norwich, UK | 01/07/2024 | PRJNA1346933 | SRR35853277 |
| PID-1150-22EPA087CP15 | *C. coli* | Cell culture | Norwich, UK | 01/07/2024 | PRJNA1346933 | SRR35853276 |
| PID-1150-22EPA087CP16 | *C. coli* | Cell culture | Norwich, UK | 01/07/2024 | PRJNA1346933 | SRR35853275 |
| PID-1150-22EPA087CP17 | *C. coli* | Cell culture | Norwich, UK | 01/07/2024 | PRJNA1346933 | SRR35853274 |
| PID-1150-22EPA087CP18 | *C. coli* | Cell culture | Norwich, UK | 01/07/2024 | PRJNA1346933 | SRR35853273 |
| PID-1150-22EPA087CP2 | *C. coli* | Cell culture | Norwich, UK | 01/07/2024 | PRJNA1346933 | SRR35853271 |
| PID-1150-22EPA087CP3 | *C. coli* | Cell culture | Norwich, UK | 01/07/2024 | PRJNA1346933 | SRR35853343 |
| PID-1150-22EPA087CP4 | *C. coli* | Cell culture | Norwich, UK | 01/07/2024 | PRJNA1346933 | SRR35853342 |
| PID-1150-22EPA087CP5 | *C. coli* | Cell culture | Norwich, UK | 01/07/2024 | PRJNA1346933 | SRR35853341 |
| PID-1150-22EPA087CP6 | *C. coli* | Cell culture | Norwich, UK | 01/07/2024 | PRJNA1346933 | SRR35853340 |
| PID-1150-22EPA087CP7 | *C. coli* | Cell culture | Norwich, UK | 01/07/2024 | PRJNA1346933 | SRR35853339 |
| PID-1150-22EPA087CP8 | *C. coli* | Cell culture | Norwich, UK | 01/07/2024 | PRJNA1346933 | SRR35853338 |
| PID-1150-22EPA087CP9 | *C. coli* | Cell culture | Norwich, UK | 01/07/2024 | PRJNA1346933 | SRR35853337 |
| PID-1150-22EPA088CP1 | *C. jejuni* | Cell culture | Norwich, UK | 01/07/2024 | PRJNA1346933 | SRR35853335 |
| PID-1150-22EPA088CP10 | *C. jejuni* | Cell culture | Norwich, UK | 01/07/2024 | PRJNA1346933 | SRR35853335 |
| PID-1150-22EPA088CP11 | *C. jejuni* | Cell culture | Norwich, UK | 01/07/2024 | PRJNA1346933 | SRR35853334 |
| PID-1150-22EPA088CP12 | *C. jejuni* | Cell culture | Norwich, UK | 01/07/2024 | PRJNA1346933 | SRR35853332 |
| PID-1150-22EPA088CP13 | *C. jejuni* | Cell culture | Norwich, UK | 01/07/2024 | PRJNA1346933 | SRR35853331 |
| PID-1150-22EPA088CP14 | *C. jejuni* | Cell culture | Norwich, UK | 01/07/2024 | PRJNA1346933 | SRR35853330 |
| PID-1150-22EPA088CP2 | *C. jejuni* | Cell culture | Norwich, UK | 01/07/2024 | PRJNA1346933 | SRR35853329 |
| PID-1150-22EPA088CP3 | *C. jejuni* | Cell culture | Norwich, UK | 01/07/2024 | PRJNA1346933 | SRR35853328 |
| PID-1150-22EPA088CP4 | *C. jejuni* | Cell culture | Norwich, UK | 01/07/2024 | PRJNA1346933 | SRR35853327 |
| PID-1150-22EPA088CP5 | *C. jejuni* | Cell culture | Norwich, UK | 01/07/2024 | PRJNA1346933 | SRR35853326 |
| PID-1150-22EPA088CP6 | *C. jejuni* | Cell culture | Norwich, UK | 01/07/2024 | PRJNA1346933 | SRR35853325 |
| PID-1150-22EPA088CP7 | *C. jejuni* | Cell culture | Norwich, UK | 01/07/2024 | PRJNA1346933 | SRR35853324 |
| PID-1150-22EPA088CP8 | *C. jejuni* | Cell culture | Norwich, UK | 01/07/2024 | PRJNA1346933 | SRR35853323 |
| PID-1150-22EPA088CP9 | *C. jejuni* | Cell culture | Norwich, UK | 01/07/2024 | PRJNA1346933 | SRR35853321 |
| PID-1250-22EPA080CP1 | *C. jejuni* | Cell culture | Norwich, UK | 01/07/2024 | PRJNA1346933 | SRR35853320 |
| PID-1250-22EPA080CP10 | *C. jejuni* | Cell culture | Norwich, UK | 01/07/2024 | PRJNA1346933 | SRR35853319 |
| PID-1250-22EPA080CP11 | *C. jejuni* | Cell culture | Norwich, UK | 01/07/2024 | PRJNA1346933 | SRR35853318 |
| PID-1250-22EPA080CP12 | *C. jejuni* | Cell culture | Norwich, UK | 01/07/2024 | PRJNA1346933 | SRR35853317 |
| PID-1250-22EPA080CP13 | *C. jejuni* | Cell culture | Norwich, UK | 01/07/2024 | PRJNA1346933 | SRR35853316 |
| PID-1250-22EPA080CP14 | *C. jejuni* | Cell culture | Norwich, UK | 01/07/2024 | PRJNA1346933 | SRR35853272 |
| PID-1250-22EPA080CP15 | *C. jejuni* | Cell culture | Norwich, UK | 01/07/2024 | PRJNA1346933 | SRR35853270 |
| PID-1250-22EPA080CP16 | *C. jejuni* | Cell culture | Norwich, UK | 01/07/2024 | PRJNA1346933 | SRR35853269 |
| PID-1250-22EPA080CP17 | *C. jejuni* | Cell culture | Norwich, UK | 01/07/2024 | PRJNA1346933 | SRR35853268 |
| PID-1250-22EPA080CP18 | *C. jejuni* | Cell culture | Norwich, UK | 01/07/2024 | PRJNA1346933 | SRR35853266 |
| PID-1250-22EPA080CP19 | *C. jejuni* | Cell culture | Norwich, UK | 01/07/2024 | PRJNA1346933 | SRR35853265 |
| PID-1250-22EPA080CP2 | *C. jejuni* | Cell culture | Norwich, UK | 01/07/2024 | PRJNA1346933 | SRR35853264 |
| PID-1250-22EPA080CP3 | *C. jejuni* | Cell culture | Norwich, UK | 01/07/2024 | PRJNA1346933 | SRR35853263 |
| PID-1250-22EPA080CP4 | *C. jejuni* | Cell culture | Norwich, UK | 01/07/2024 | PRJNA1346933 | SRR35853262 |
| PID-1250-22EPA080CP5 | *C. jejuni* | Cell culture | Norwich, UK | 01/07/2024 | PRJNA1346933 | SRR35853261 |
| PID-1250-22EPA080CP6 | *C. jejuni* | Cell culture | Norwich, UK | 01/07/2024 | PRJNA1346933 | SRR35853260 |
| PID-1250-22EPA080CP7 | *C. jejuni* | Cell culture | Norwich, UK | 01/07/2024 | PRJNA1346933 | SRR35853259 |
| PID-1250-22EPA080CP8 | *C. jejuni* | Cell culture | Norwich, UK | 01/07/2024 | PRJNA1346933 | SRR35853258 |
| PID-1250-22EPA080CP9 | *C. jejuni* | Cell culture | Norwich, UK | 01/07/2024 | PRJNA1346933 | SRR35853257 |
| PID-1250-22EPA083CP1 | *C. jejuni* | Cell culture | Norwich, UK | 01/06/2022 | PRJNA1346933 | SRR35853254 |
| PID-1250-22EPA083CP10 | *C. jejuni* | Cell culture | Norwich, UK | 01/06/2022 | PRJNA1346933 | SRR35853254 |
| PID-1250-22EPA083CP11 | *C. jejuni* | Cell culture | Norwich, UK | 01/06/2022 | PRJNA1346933 | SRR35853253 |
| PID-1250-22EPA083CP12 | *C. jejuni* | Cell culture | Norwich, UK | 01/06/2022 | PRJNA1346933 | SRR35853252 |
| PID-1250-22EPA083CP13 | *C. jejuni* | Cell culture | Norwich, UK | 01/06/2022 | PRJNA1346933 | SRR35853251 |
| PID-1250-22EPA083CP14 | *C. jejuni* | Cell culture | Norwich, UK | 01/06/2022 | PRJNA1346933 | SRR35853250 |
| PID-1250-22EPA083CP15 | *C. jejuni* | Cell culture | Norwich, UK | 01/06/2022 | PRJNA1346933 | SRR35853249 |
| PID-1250-22EPA083CP16 | *C. jejuni* | Cell culture | Norwich, UK | 01/06/2022 | PRJNA1346933 | SRR35853248 |
| PID-1250-22EPA083CP17 | *C. jejuni* | Cell culture | Norwich, UK | 01/06/2022 | PRJNA1346933 | SRR35853247 |
| PID-1250-22EPA083CP18 | *C. jejuni* | Cell culture | Norwich, UK | 01/06/2022 | PRJNA1346933 | SRR35853246 |
| PID-1250-22EPA083CP19 | *C. jejuni* | Cell culture | Norwich, UK | 01/06/2022 | PRJNA1346933 | SRR35853244 |
| PID-1250-22EPA083CP2 | *C. jejuni* | Cell culture | Norwich, UK | 01/06/2022 | PRJNA1346933 | SRR35853159 |
| PID-1250-22EPA083CP20 | *C. jejuni* | Cell culture | Norwich, UK | 01/06/2022 | PRJNA1346933 | SRR35853158 |
| PID-1250-22EPA083CP3 | *C. jejuni* | Cell culture | Norwich, UK | 01/06/2022 | PRJNA1346933 | SRR35853157 |
| PID-1250-22EPA083CP4 | *C. jejuni* | Cell culture | Norwich, UK | 01/06/2022 | PRJNA1346933 | SRR35853156 |
| PID-1250-22EPA083CP5 | *C. jejuni* | Cell culture | Norwich, UK | 01/06/2022 | PRJNA1346933 | SRR35853155 |
| PID-1250-22EPA083CP6 | *C. jejuni* | Cell culture | Norwich, UK | 01/06/2022 | PRJNA1346933 | SRR35853154 |
| PID-1250-22EPA083CP7 | *C. jejuni* | Cell culture | Norwich, UK | 01/06/2022 | PRJNA1346933 | SRR35853153 |
| PID-1250-22EPA083CP8 | *C. jejuni* | Cell culture | Norwich, UK | 01/06/2022 | PRJNA1346933 | SRR35853152 |
| PID-1250-22EPA083CP9 | *C. jejuni* | Cell culture | Norwich, UK | 01/06/2022 | PRJNA1346933 | SRR35853151 |
| PID-1291-22EPA077CP1 | *C. jejuni* | Cell culture | Norwich, UK | 01/06/2022 | PRJNA1346933 | SRR35853149 |
| PID-1291-22EPA077CP10 | *C. jejuni* | Cell culture | Norwich, UK | 01/06/2022 | PRJNA1346933 | SRR35853148 |
| PID-1291-22EPA077CP2 | *C. jejuni* | Cell culture | Norwich, UK | 01/06/2022 | PRJNA1346933 | SRR35853147 |
| PID-1291-22EPA077CP3 | *C. jejuni* | Cell culture | Norwich, UK | 01/06/2022 | PRJNA1346933 | SRR35853146 |
| PID-1291-22EPA077CP4 | *C. jejuni* | Cell culture | Norwich, UK | 01/06/2022 | PRJNA1346933 | SRR35853145 |
| PID-1291-22EPA077CP5 | *C. jejuni* | Cell culture | Norwich, UK | 01/06/2022 | PRJNA1346933 | SRR35853144 |
| PID-1291-22EPA077CP6 | *C. jejuni* | Cell culture | Norwich, UK | 01/06/2022 | PRJNA1346933 | SRR35853143 |
| PID-1291-22EPA077CP7 | *C. jejuni* | Cell culture | Norwich, UK | 01/06/2022 | PRJNA1346933 | SRR35853142 |
| PID-1291-22EPA077CP8 | *C. jejuni* | Cell culture | Norwich, UK | 01/06/2022 | PRJNA1346933 | SRR35853141 |
| PID-1291-22EPA077CP9 | *C. jejuni* | Cell culture | Norwich, UK | 01/06/2022 | PRJNA1346933 | SRR35853140 |
| PID-2029-24EPA149CP01 | *C. coli* | Cell culture | Norwich, UK | 01/09/2024 | PRJNA1346933 | SRR35853138 |
| PID-2029-24EPA149CP02 | *C. coli* | Cell culture | Norwich, UK | 01/09/2024 | PRJNA1346933 | SRR35853137 |
| PID-2029-24EPA149CP03 | *C. coli* | Cell culture | Norwich, UK | 01/09/2024 | PRJNA1346933 | SRR35853136 |
| PID-2029-24EPA150CP01 | *C. jejuni* | Cell culture | Norwich, UK | 01/09/2024 | PRJNA1346933 | SRR35853135 |
| PID-2029-24EPA150CP02 | *C. jejuni* | Cell culture | Norwich, UK | 01/09/2024 | PRJNA1346933 | SRR35853134 |
| PID-2029-24EPA150CP03 | *C. jejuni* | Cell culture | Norwich, UK | 01/09/2024 | PRJNA1346933 | SRR35853133 |
| PID-2029-24EPA150CP04 | *C. jejuni* | Cell culture | Norwich, UK | 01/09/2024 | PRJNA1346933 | SRR35853132 |
| PID-2029-24EPA150CP05 | *C. jejuni* | Cell culture | Norwich, UK | 01/09/2024 | PRJNA1346933 | SRR35853243 |
| PID-2029-24EPA150CP06 | *C. jejuni* | Cell culture | Norwich, UK | 01/09/2024 | PRJNA1346933 | SRR35853242 |
| PID-2029-24EPA150CP07 | *C. jejuni* | Cell culture | Norwich, UK | 01/09/2024 | PRJNA1346933 | SRR35853241 |
| PID-2029-24EPA152CP01 | *C. jejuni* | Cell culture | Norwich, UK | 01/09/2024 | PRJNA1346933 | SRR35853239 |
| PID-2029-24EPA152CP02 | *C. jejuni* | Cell culture | Norwich, UK | 01/09/2024 | PRJNA1346933 | SRR35853238 |
| PID-2029-24EPA152CP03 | *C. jejuni* | Cell culture | Norwich, UK | 01/09/2024 | PRJNA1346933 | SRR35853237 |
| PID-2029-24EPA152CP04 | *C. jejuni* | Cell culture | Norwich, UK | 01/09/2024 | PRJNA1346933 | SRR35853236 |
| PID-2029-24EPA152CP05 | *C. jejuni* | Cell culture | Norwich, UK | 01/09/2024 | PRJNA1346933 | SRR35853235 |
| PID-2029-24EPA152CP06 | *C. jejuni* | Cell culture | Norwich, UK | 01/09/2024 | PRJNA1346933 | SRR35853234 |
| PID-2029-24EPA152CP07 | *C. jejuni* | Cell culture | Norwich, UK | 01/09/2024 | PRJNA1346933 | SRR35853233 |
| PID-2029-24EPA152CP08 | *C. jejuni* | Cell culture | Norwich, UK | 01/09/2024 | PRJNA1346933 | SRR35853232 |
| PID-2029-24EPA152CP09 | *C. jejuni* | Cell culture | Norwich, UK | 01/09/2024 | PRJNA1346933 | SRR35853231 |
| PID-2029-24EPA152CP10 | *C. jejuni* | Cell culture | Norwich, UK | 01/09/2024 | PRJNA1346933 | SRR35853230 |
| PID-2029-24EPA153CP01 | *C. jejuni* | Cell culture | Norwich, UK | 01/09/2024 | PRJNA1346933 | SRR35853228 |
| PID-2029-24EPA153CP02 | *C. jejuni* | Cell culture | Norwich, UK | 01/09/2024 | PRJNA1346933 | SRR35853227 |
| PID-2029-24EPA153CP03 | *C. jejuni* | Cell culture | Norwich, UK | 01/09/2024 | PRJNA1346933 | SRR35853226 |
| PID-2029-24EPA153CP04 | *C. jejuni* | Cell culture | Norwich, UK | 01/09/2024 | PRJNA1346933 | SRR35853225 |
| PID-2029-24EPA153CP06 | *C. jejuni* | Cell culture | Norwich, UK | 01/09/2024 | PRJNA1346933 | SRR35853224 |
| PID-2029-24EPA153CP07 | *C. jejuni* | Cell culture | Norwich, UK | 01/09/2024 | PRJNA1346933 | SRR35853223 |
| PID-2029-24EPA153CP08 | *C. jejuni* | Cell culture | Norwich, UK | 01/09/2024 | PRJNA1346933 | SRR35853222 |
| PID-2029-24EPA153CP09 | *C. jejuni* | Cell culture | Norwich, UK | 01/09/2024 | PRJNA1346933 | SRR35853221 |
| PID-2029-24EPA153CP10 | *C. jejuni* | Cell culture | Norwich, UK | 01/09/2024 | PRJNA1346933 | SRR35853220 |
| PID-2029-24EPA154CP01 | *C. jejuni* | Cell culture | Norwich, UK | 01/09/2024 | PRJNA1346933 | SRR35853219 |
| PID-2029-24EPA154CP02 | *C. jejuni* | Cell culture | Norwich, UK | 01/09/2024 | PRJNA1346933 | SRR35853216 |
| PID-2029-24EPA154CP03 | *C. jejuni* | Cell culture | Norwich, UK | 01/09/2024 | PRJNA1346933 | SRR35853315 |
| PID-2029-24EPA154CP04 | *C. jejuni* | Cell culture | Norwich, UK | 01/09/2024 | PRJNA1346933 | SRR35853314 |
| PID-2029-24EPA154CP06 | *C. jejuni* | Cell culture | Norwich, UK | 01/09/2024 | PRJNA1346933 | SRR35853313 |
| PID-2029-24EPA154CP07 | *C. jejuni* | Cell culture | Norwich, UK | 01/09/2024 | PRJNA1346933 | SRR35853312 |
| PID-2029-24EPA154CP08 | *C. jejuni* | Cell culture | Norwich, UK | 01/09/2024 | PRJNA1346933 | SRR35853311 |
| PID-2029-24EPA154CP09 | *C. jejuni* | Cell culture | Norwich, UK | 01/09/2024 | PRJNA1346933 | SRR35853310 |
| PID-2029-24EPA154CP10 | *C. jejuni* | Cell culture | Norwich, UK | 01/09/2024 | PRJNA1346933 | SRR35853309 |
| PID-2029-24EPA155CP01 | *C. jejuni* | Cell culture | Norwich, UK | 01/09/2024 | PRJNA1346933 | SRR35853308 |
| PID-2029-24EPA155CP02 | *C. jejuni* | Cell culture | Norwich, UK | 01/09/2024 | PRJNA1346933 | SRR35853307 |
| PID-2029-24EPA155CP03 | *C. jejuni* | Cell culture | Norwich, UK | 01/09/2024 | PRJNA1346933 | SRR35853305 |
| PID-2029-24EPA155CP04 | *C. jejuni* | Cell culture | Norwich, UK | 01/09/2024 | PRJNA1346933 | SRR35853304 |
| PID-2029-24EPA155CP05 | *C. jejuni* | Cell culture | Norwich, UK | 01/09/2024 | PRJNA1346933 | SRR35853303 |
| PID-2029-24EPA155CP06 | *C. jejuni* | Cell culture | Norwich, UK | 01/09/2024 | PRJNA1346933 | SRR35853302 |
| PID-2029-24EPA155CP07 | *C. jejuni* | Cell culture | Norwich, UK | 01/09/2024 | PRJNA1346933 | SRR35853301 |
| PID-2029-24EPA155CP08 | *C. jejuni* | Cell culture | Norwich, UK | 01/09/2024 | PRJNA1346933 | SRR35853300 |
| PID-2029-24EPA155CP09 | *C. jejuni* | Cell culture | Norwich, UK | 01/09/2024 | PRJNA1346933 | SRR35853299 |
| PID-2029-24EPA155CP10 | *C. jejuni* | Cell culture | Norwich, UK | 01/09/2024 | PRJNA1346933 | SRR35853298 |
| PID-2029-24EPA156CP01 | *C. jejuni* | Cell culture | Norwich, UK | 01/09/2024 | PRJNA1346933 | SRR35853297 |
| PID-2029-24EPA156CP03 | *C. jejuni* | Cell culture | Norwich, UK | 01/09/2024 | PRJNA1346933 | SRR35853296 |
| PID-2029-24EPA156CP04 | *C. jejuni* | Cell culture | Norwich, UK | 01/09/2024 | PRJNA1346933 | SRR35853294 |
| PID-2029-24EPA156CP06 | *C. jejuni* | Cell culture | Norwich, UK | 01/09/2024 | PRJNA1346933 | SRR35853293 |
| PID-2029-24EPA156CP07 | *C. jejuni* | Cell culture | Norwich, UK | 01/09/2024 | PRJNA1346933 | SRR35853292 |
| PID-2029-24EPA156CP08 | *C. jejuni* | Cell culture | Norwich, UK | 01/09/2024 | PRJNA1346933 | SRR35853291 |
| PID-2029-24EPA156CP09 | *C. jejuni* | Cell culture | Norwich, UK | 01/09/2024 | PRJNA1346933 | SRR35853290 |
| PID-2029-24EPA156CP10 | *C. jejuni* | Cell culture | Norwich, UK | 01/09/2024 | PRJNA1346933 | SRR35853289 |
| PID-2029-24EPA158CP01 | *C. jejuni* | Cell culture | Norwich, UK | 01/09/2024 | PRJNA1346933 | SRR35853288 |
| PID-2029-24EPA158CP02 | *C. jejuni* | Cell culture | Norwich, UK | 01/09/2024 | PRJNA1346933 | SRR35853131 |
| PID-2029-24EPA158CP03 | *C. jejuni* | Cell culture | Norwich, UK | 01/09/2024 | PRJNA1346933 | SRR35853130 |
| PID-2029-24EPA159CP01 | *C. jejuni* | Cell culture | Norwich, UK | 01/09/2024 | PRJNA1346933 | SRR35853129 |
| PID-2029-24EPA159CP02 | *C. jejuni* | Cell culture | Norwich, UK | 01/09/2024 | PRJNA1346933 | SRR35853127 |
| PID-2029-24EPA159CP03 | *C. jejuni* | Cell culture | Norwich, UK | 01/09/2024 | PRJNA1346933 | SRR35853126 |
| PID-2029-24EPA159CP04 | *C. jejuni* | Cell culture | Norwich, UK | 01/09/2024 | PRJNA1346933 | SRR35853125 |
| PID-2029-24EPA159CP05 | *C. jejuni* | Cell culture | Norwich, UK | 01/09/2024 | PRJNA1346933 | SRR35853124 |
| PID-2029-24EPA159CP06 | *C. jejuni* | Cell culture | Norwich, UK | 01/09/2024 | PRJNA1346933 | SRR35853123 |
| PID-2029-24EPA161CP01 | *C. jejuni* | Cell culture | Norwich, UK | 01/09/2024 | PRJNA1346933 | SRR35853122 |
| PID-2029-24EPA161CP03 | *C. jejuni* | Cell culture | Norwich, UK | 01/09/2024 | PRJNA1346933 | SRR35853121 |
| PID-2029-24EPA161CP04 | *C. jejuni* | Cell culture | Norwich, UK | 01/09/2024 | PRJNA1346933 | SRR35853120 |
| PID-2029-24EPA161CP05 | *C. jejuni* | Cell culture | Norwich, UK | 01/09/2024 | PRJNA1346933 | SRR35853119 |
| PID-2029-24EPA161CP06 | *C. jejuni* | Cell culture | Norwich, UK | 01/09/2024 | PRJNA1346933 | SRR35853118 |
| PID-2029-24EPA161CP07 | *C. jejuni* | Cell culture | Norwich, UK | 01/09/2024 | PRJNA1346933 | SRR35853116 |
| PID-2029-24EPA161CP08 | *C. jejuni* | Cell culture | Norwich, UK | 01/09/2024 | PRJNA1346933 | SRR35853115 |
| PID-2029-24EPA161CP09 | *C. jejuni* | Cell culture | Norwich, UK | 01/09/2024 | PRJNA1346933 | SRR35853114 |
